# Efficient photosynthesis and economic water use of citrus leaves depend on hybrid, cultivar and leaf type

**DOI:** 10.3389/fpls.2025.1536703

**Published:** 2025-09-04

**Authors:** Mingjiong Zhao, Zhenshan Liu, Yanping Hu, Shilai Yi, Yueqiang Zhang, Bin Hu, Xiaojun Shi, Heinz Rennenberg

**Affiliations:** ^1^ Center of Molecular Ecophysiology (CMEP), College of Resources and Environment, Southwest University, Chongqing, China; ^2^ Citrus Research Institute, Chinese Academy of Agricultural Sciences, Chongqing, China; ^3^ Interdisciplinary Research Center for Agriculture Green Development in Yangtze River Basin, College of Resources and Environment, Southwest University, Chongqing, China

**Keywords:** citrus hybrid, cultivar, gene expression, leaf type, photosynthesis, water use efficiency, stomatal conductance, transpiration

## Abstract

Efficient photosynthesis and economic water use are essential for citrus growth, development and fruit production. The present study was aimed to characterize these processes in current-year spring, autumn, summer and spring shoots of citrus hybrid OP with cultivars ‘Orah’ (OR) and ‘Ponkan’ (PO) and citrus hybrid NT with cultivars ‘Newhall navel orange’ (NO) and ‘Tarocco’ (TA). Cultivars NO and PO show mid-fruit ripening, and cultivars TA and OR late-fruit ripening under field conditions. To characterize photosynthesis and water use, CO_2_ and H_2_O gas exchange, water use efficiency and expression of related genes were analyzed. The CO_2_ and H_2_O gas exchange parameters measured were determined by hybrid, cultivar and leaf type. Genes involved in lipid and pectin catabolic processes, cell wall biogenesis and modification, carbohydrate and xyloglucan metabolism, cellulose biosynthesis and cell growth were significantly upregulated in current-year spring shoots compared to the other leaf types investigated. Expression of photosynthesis- and transpiration-related genes was significantly enhanced in leaves of late-ripening cultivar OR compared to the other cultivars. These results indicate that the two hybrids of the four citrus cultivars studied differ in the expression of photosynthesis- and transpiration-related genes, but these differences cannot be attributed to fruit maturation.

## 1 Introduction

Citrus is an evergreen plant genus of the family *Rutaceae*, mostly belonging to the subfamily *Aurantioideae* (Bayer et al., 2009). It includes one of the most important groups of fruit trees with high economic value and is widely planted all over the world (Xiong et al., 2021). In South China, the planting area of citrus covers approximately 2.73 × 10^6^ ha, and its annual fruit production amounts to more than 4.30 × 10^7^ tons (Tu et al., 2021). In citrus, three types of shoots are produced during the growing season. The main type of spring shoots grows in late winter or early spring, and two additional types of summer shoots and autumn shoots grow at the end of June and late September (Li et al., 2010). Spring shoots play the most important role in determining the growth and yield of citrus (Zhang et al., 2011). Summer shoots, especially those located at the top of the tree, mostly become vigorous, nutrient-consuming, non-productive, and attractive to several pests. Therefore, regulation of the summer shoot numbers is required in citrus orchards (Arenas-Arenas et al., 2021). By contrast, autumn shoots with excellent nutrition and growth are retained because they constitute mother branches for future fruits (Verreynne and Lovatt, 2009). Based on these different functions, differences between these types of shoots are to be expected at the physiological and molecular level. This is also demonstrated by Xiong et al. (2020) by showing that net photosynthesis is higher in citrus leaves of summer shoots compared to spring shoots. In broad-leaved evergreen tea trees, different leaf types showed differential expression of genes involved in processes like cell cycle regulation, starch and sucrose metabolism, photosynthesis, phenylpropanoid and flavonoid biosynthesis (Guo et al., 2017). Similar information at the molecular level is not available for different citrus leaf types.

As one of the most important physiological functions of plant leaves, photosynthesis is not only vital for plant growth and development but also for fruit production (Niu et al., 2008; Evans, 2013; Flexas and Carriquí, 2020). It determines the productivity of plants, participates in nutrient flow and cycling in the ecosystem, and is the main pathway for plant carbon sequestration (Ashraf and Harris, 2013). In this context, photosynthesis of citrus leaves is considered to constitute the main source of carbon and energy required for both growth and fruit production (Ribeiro and MaChado, 2007). The latter is indicated by the observation that carbohydrates stored in mature citrus leaves are depleted during the initial stages of budding and flowering and subsequent fruit development is supported by the actual photosynthesis of adjacent leaves (Syvertsen and Lloyd, 1994; Nebauer et al., 2011).

Apart from photosynthesis, foliar water use efficiency (WUE), as a key measure for the consumption of water resources, is an important target for crop selection and breeding under changing environmental conditions (Linderson et al., 2012; Gago et al., 2014; Tomás et al., 2014; IPCC, 2021). In short-term analyses, intrinsic water use efficiency (WUEi) is usually differentiated from instantaneous water use efficiency (WUEinst) (Fischer and Turner, 1978). WUEi and WUEinst are frequently used to characterize genetic differences and environmental impacts on foliar gas exchange (Chaves and Oliveira, 2004; Flexas et al., 2008; Galmés et al., 2007; Morison et al., 2008). For WUE, Tr (transpiration) controlled by Gs (stomatal conductance) plays a crucial role, since it affects not only the water balance of plants, but also numerous other important physiological processes and, hence, growth and productivity of fruit trees (Lo Gullo et al., 2003; Solari et al., 2006). The stomata are the vital channels for both H_2_O and CO_2_ gas exchange between plants and the atmosphere. They control photosynthetic carbon fixation as well as water loss by adjustment of the degree of opening (Berry et al., 2010; Lin Y. et al., 2015; Wolf et al., 2016; Dewar et al., 2018). Therefore, stomatal conductance also affects citrus fruit production and fruit quality (Wagner et al., 2021). As a consequence, understanding photosynthesis and WUE is essential for the selection of cultivars/hybrids adapted in growth and fruit production to the particular environment of cultivation, especially under water-limited conditions. Such conditions already increased in duration, frequency and severity due to global climate change in the past and are supposed to further increase in future (Gao et al., 2016; Jia et al., 2024).

Photosynthesis and WUE have been researched in numerous plant species including evergreen trees both, at the physiological and transcriptional level. Most studies on gas exchange by evergreen trees are based on stress exposure either in nurseries or under natural conditions in forests, indicating that heterogenic photosynthesis occurs during severe drought as result of patchy stomatal closure (Guardia et al., 2012). However, different evergreen species differ in the response of gas exchange to stress. For example, on an annual basis, live oak assimilates more CO_2_, but also loses significantly more water through transpiration than juniper under semiarid conditions (Bendevis et al., 2010). In addition, the response of water use efficiency to stress depends of the cultivar of the species analyzed. For example, ponderosa pine seedlings from warm environments show slower growth and higher water-use efficiency than seedlings from cool environments (Dixit et al., 2022). Also, photosynthesis and WUE in evergreen broad-leaved citrus have been studied in the context of irrigation and drought (Wagner et al., 2021). Photosynthesis was shown to not only depend on citrus leaf type but also on citrus hybrid and cultivar (Arbona et al., 2009; Sang et al., 2015; Xiong et al., 2020). For instance, the latest research indicates that under drought and control conditions, Newhall navel orange exhibit better physiological performance (increased Pn in leaves) compared with Orah (Jia et al., 2024). Whether the dependency on hybrid or cultivar is a consequence of different times of fruit ripening and connected seasonal differences in the requirement of enhanced allocation of carbohydrates from the leaves to the fruits (Stander et al., 2018) has so far not been elucidated. Massive closure of stomata was observed in citrus leaves of different genotypes upon flooding associated with a decrease in Tr, Gs and WUE (Arbona et al., 2009). However, differences in the performance of photosynthesis and WUE of citrus leaves between leaf types, hybrids and cultivars with different fruit ripening periods have not been reported at both, the physiological and transcriptional level. Still, this information is essential for a better understanding of the key processes determining the quality of citrus fruits that largely depends on the allocation of carbohydrates from the leaves to the fruits. Therefore, further information on photosynthesis and WUE is required at the physiological and molecular level in different leaf (shoot) types of citrus hybrids and cultivars.

The present study was aimed to characterize the efficiency of photosynthesis and foliar WUE of various leaf types in citrus hybrids and cultivars differing in fruit maturation. For this purpose, we combined CO_2_ and H_2_O gas exchange and WUE measurements with gene expression analysis in leaves on spring, summer, and autumn shoots of four citrus cultivars. We hypothesized that (i) photosynthesis and WUE of the citrus leaves are significantly affected by hybrid, cultivar and leaf type; (ii) these effects can be related to fruit maturation that relies on photosynthetic carbohydrate production, and (iii) are determined by gene expression.

## 2 Materials and methods

### 2.1 Plant materials

The experiments were performed in 2021 at the Citrus Research Institute of the Chinese Academy of Agricultural Sciences (29°45′N, 106°22′E) in the Beibei District of Chongqing. Four cultivars (mature, ca. 10 years old citrus trees) were selected for the experiments, *i.e.*, the mid-ripening cultivars ‘Newhall navel orange’ (NO) of *Citrus sinensis* (L.) Osbeck and ‘Ponkan’ (PO) of *Citrus reticulata* Blanco(which are harvested in December), and the late-ripening cultivars ‘Tarocco’ (TA) of *Citrus sinensis* (L.) Osbeck and ‘Orah’ (OR) of *Citrus reticulata* Blanco (https://cfh.ac.cn) (which are harvested in March). The sweet orange cultivars NO and TA are combined as hybrid NT, while the loose-skin citrus cultivars OR and PO are combined as hybrid OP.

All citrus cultivars were grafted on *Poncirus trifoliata* rootstocks, planted under the same field management practices with a spacing of 3m x 4m, and maintained under the same fertilization conditions. The mean annual temperature was 19.3 °C, annual sunshine time was 1179 h, and annual precipitation was 1172 mm with maximum temperature in July. The orchard soil was classified as loose loam with a pH value of 5.03 ± 0.29. The organic matter and available N, P, and K of the orchard soil were 20.71 ± 2.86 g·kg^−1^, 89.15 ± 4.59, 49.61 ± 7.64, and 190.28 ± 10.07 mg·kg^−1^, respectively (Wan et al., 2021). The trees of *Citrus* were watered 15 times annually, pruned at the end of February each year and sprayed with pesticides 5 times per year to control diseases and pests. In mid-March, 5.0 kg oil cake fertilizer and 1.0 kg mineral fertilizer (N: P_2_O_5_: K_2_O= 21:8:11) were applied per plant, in mid-June, 1.0 kg mineral fertilizer (N: P_2_O_5_: K_2_O= 12:12:21) was added per plant to the soil, and 5.0 kg organic fertilizer (cow dung) was applied per plant in late October each year. *Citrus* trees were fertilized every year according to this schedule.

### 2.2 Experimental design and plant tissue collection

The experiments adopted a completely randomized block design with three factors, *i.e.*, hybrid (OP/NT), cultivar (OR, PO, NO/TA) and leaf type at four replicates of the citrus tree (n=4) (see 2.1 for details). Four leaf types were selected for sampling and the analyses of CO_2_ and H_2_O gas exchange, including spring-, summer- and autumn shoots of 2020, and new flushed current-year spring shoot leaves of 2021. The spring shoots have short leaf internodes, small leaf shape, thin front end, and narrow tail; the summer shoots are large and thick, the wing lobes are large or distinct, and the ends are blunt; the autumn shoots’ leaf size is between spring and summer shoots. For each citrus cultivar and hybrid, four tree replicates of uniform growth were selected (n=4). On each of the four tree replicates, CO_2_ and H_2_O gas exchange was measured on four healthy, fully expanded sun-facing unshaded leaves of each leaf type at each leaf age (n=4). For RNA-seq analyses, 1 to 2 g fresh weight of each leaf type and age were collected in three replicates from each cultivar and hybrid in April 2021 (n=3), placed in 10 mL centrifuge tubes, immediately frozen in liquid nitrogen, and stored at -80 °C until further analyses. Plant material was homogenized in liquid N_2_ to a powder using a fully automated refrigeration grinder (JXFSTPRP-CL, Jingxin Ltd., Shanghai, China).

### 2.3 CO_2_ and H_2_O gas exchange and water use efficiency analyses

On April 21 (14-24C°, sunny) and 22 (9-17C°, cloudy), 2021, gas exchange parameters were determined from 9 a.m to 12:00 am each day by a portable Li-6800 photosynthesis system (LI-COR, Lincoln, Nebraska, USA). During the measurements, photosynthetic active radiation (PAR), CO_2_ concentration, temperature, and relative humidity (RH) in the leaf chamber were set at 1500 μmol/m_2_·s, 400 μmol/mol, 25 °C, and 60%, respectively. Before the parameters were logged, equilibration of leaves depended on the time required for stabilization of the parameters and varied between 0 and 1 to 3 min. During the measurements the parameters measured were stable. The parameters measured included net rates of photosynthetic CO_2_ fixation (Pn), transpiration (Tr), stomatal conductance (Gs) and intracellular CO_2_ concentration (Ci). The intrinsic water use efficiency was calculated as follow: WUEi = Pn/Gs; and instantaneous water use efficiency was calculated as follow: WUEinst = Pn/Tr (Fischer and Turner, 1978). All measurements were repeated 4 times for each leaf on each plant of each cultivar.

### 2.4 Transcriptome analysis

In April 2021, three replicate samples of each leaf type of the four citrus cultivars were collected and immediately frozen in liquid nitrogen. Total RNA from 48 leaf samples was extracted for RNA-sequencing using the RNeasy Plant Mini Kit (Qiagen, Hilden, Germany https://www.qiagen.com) according to the manufacturer’s instructions. DNA contamination was removed from the samples by DNAse digestion, and the DNAse-treated samples were polyA-enriched using oligodT dynabeads (Invitrogen, Carlsbad, California, USA). Confirmation of rRNA removal and sample quantification were performed with Qubit (Thermo Fisher, Waltham, Massachusetts, USA) and an Agilent 4200 Tapestation (Agilent Technologies Inc., California, USA), respectively. All samples were prepared together to minimize batch effects.

The extracted RNA of the 48 samples was sequenced on MinION (Oxford Nanopore Technologies, Oxford, UK) using R9.4 flow cells with the relevant MinKNOW script to generate fast5 files. All fast5 reads were base-called using Guppy (https://nanoporetech.com/) to yield fastq files. NanoFilt v2.8.0 (De Coster et al., 2018) was used for filtering and trimming of reads < 300 bp. FLAIR (Tang et al., 2020) was applied to summarize the clean reads into isoforms in four main steps: alignment, correction, collapsing and quantifying. We used minimap2 (Li, 2021) to align read sequences from all samples to the C. sinensis reference genome (Xu et al., 2013). The genome alignments were performed using the splice-aware mode of minimap2 -ax splice -k 14 -uf –secondary=no as recommended (Tang et al., 2020). The correction and collapsing step of FLAIR were applied with default parameters, while Salmon (Patro et al., 2017) was used to estimate transcript and gene abundances for ONT libraries. Identification of differentially expressed genes (DEGs) was performed using DESeq2 (Love et al., 2014) at log2FC > 2 and Benjamini-Hochberg FDR-adjusted *p* values (< 0.05). Kyoto Encyclopedia of Genes and Genomes (KEGG) and Gene Ontology (GO) term enrichment in DEGs was used with clusterProfiler (Wu et al., 2021) and the significance of enrichment was estimated by Benjamini-Hochberg FDR correction (Benjamini and Hochberg, 1995). We examined the expression pattern of photosynthesis- and transpiration-related genes from DEG sets generated from a comparison between leaf types and cultivars.

### 2.5 Statistical analysis

Differences between leaf types autumn (au), summer (su) and spring shoots (sp) of citrus in CO_2_ and H_2_O gas exchange parameters were analyzed by one-way analysis of variance (ANOVA) followed by least-square significant difference (LSD) analyses at a significance level of *p* < 0.05. Data failing to match normal distribution after log_10_ transformation were subjected to Kruskal-Wallis ANOVA. Differences between leaf types current-year spring (css) and spring shoots (sp), hybrids and cultivars in CO_2_ and H_2_O gas exchange parameters were analyzed by t-test at a significance level of *p* < 0.05. The SPSS 26.0 software was used (SPSS Inc., Chicago, IL, USA) for data analysis. Figures were generated using Sigmaplot 14.0 (Systat Software, Erkrath, Germany). For Partial Least Squares-Discriminant Analysis (PLS-DA), data were processed online using the MetaboAnalyst 4.0 software (http://www.metaboanalyst.ca) (Chong et al., 2018).

## 3 Results

### 3.1 PLS-DA and transcriptomic analysis reveal the dependency of foliar gas exchange and WUE of citrus on hybrid, cultivar and leaf type

**Figure 1:**
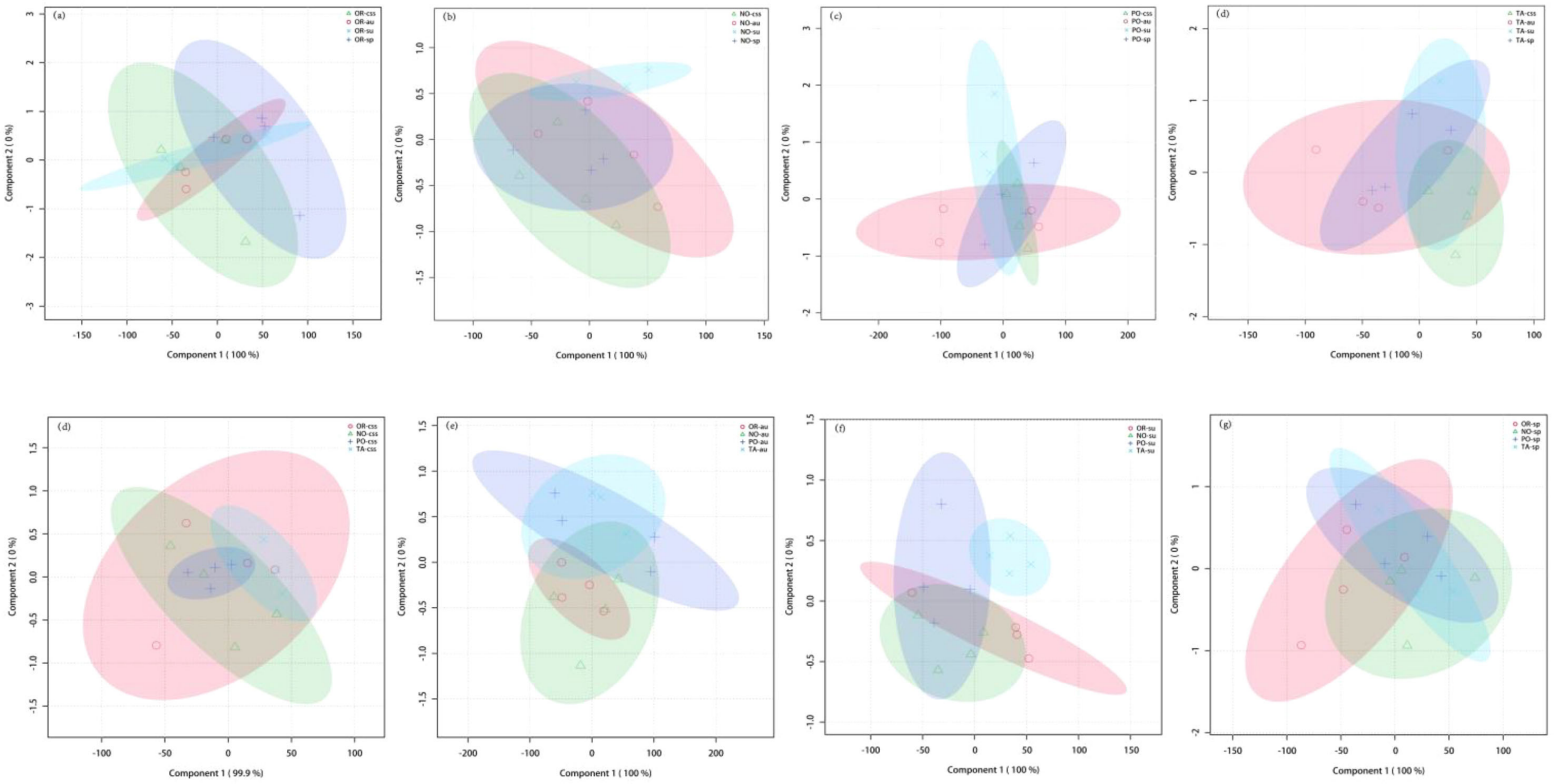
Partial least squares-discriminant analysis (PLS-DA) scores of gas exchange parameters of different leaf types, hybrids, and cultivars. **(A–D)** show the differences among current-year spring (css), autumn (au), summer (su) and spring shoots (sp) in the four cultivars Orah (OR), Newhall navel orange (NO), Ponkan (PO), and Tarocco (TA). **(E–H)** show the differences between the two hybrids and cultivars of Orah (OR), Newhall navel orange (NO), Ponkan (PO), and Tarocco (TA) in each leaf type including current-year spring (css), autumn (au), summer (su) and spring shoots (sp). The PLS-DA loading charts contain the parameters Pn (net photosynthesis), Tr (transpiration), Gs (stomatal conductance), Ci (intracellular CO_2_ concentration), WUEi (intrinsic water use efficiency), and WUEinst (instantaneous water use efficiency). The ellipses indicate the 95% confidence range.

By employing multivariate analysis on the overall 6 foliar CO_2_ and H_2_O gas exchange and WUE traits of citrus studied, partial least square-discriminant analysis (PLS-DA) revealed distinct clustering patterns between hybrids and cultivars but not between leaf types (**Supplementary Figure S1**, **Figure 1**). For different hybrids, in the summer shoots, the score plots revealed distinct clustering patterns between the hybrids OP (cultivar OR) and NT (cultivar TA) (**Figure 1F**). For different cultivars, in the summer shoots, the score plots revealed distinct clustering patterns between the mid-ripening cultivar NO and the late-ripening cultivar TA (**Figure 1F**). For different leaf types, the score plots did not reveal distinct clustering patterns (**Supplementary Figure S1**, **Figure 1**). Thus, the clustering of foliar CO_2_ and H_2_O gas exchange as well as WUE traits was generally determined by hybrids and cultivars.

To elucidate the molecular mechanisms that caused physiological differences of CO_2_ and H_2_O gas exchange and WUE between hybrids and cultivars, transcriptome analyses were performed. We detected 944 expressed photosynthesis-related genes, with 418 involved in photosynthetic electron transport, 59 participating in carbon fixation via the Calvin-Benson cycle and 467 supposed to control leaf water status via regulation of stomatal movement. An overview of gene expression variation across hybrids, cultivars and leaf types showed that the expression pattern of these genes did not reveal strictly clustering (**Supplementary Figure S2**). This result indicates that molecular differences in photosynthesis and transpiration between hybrids and cultivars were not generally related to differences in gene expression. In order to identify potential specific relationships, individual parameters of foliar gas exchange and WUE were compared with transcriptomic signatures.

To elucidate if differences in fruit maturation influence the expression of photosynthesis- and transpiration-related genes, mid-ripening (*i.e.*, NO and PO) and late-ripening (*i.e.*, OR and TA) cultivars were compared across leaf types. We discovered 29, 24, 26 and 74 DEGs between mid-ripening and late-ripening cultivars at spring, summer, autumn and current-year spring shoots, respectively (**Supplementary Tables S1-4**). These results suggest that newly developing leaves show the most distinguishing expression pattern between cultivars differing in fruit maturation. Only two genes (Cs1g12660 and Cs7g03150) were shared between these four DEG sets. Cs1g12660 is supposed to take part in the methylation pathway of lignin biosynthesis, while Cs7g03150 constitutes a multifunctional gene involved in the responses to abiotic stress such as salt, cold, and heat as well as biotic stress via regulation of abscisic acid biosynthesis. Photosynthesis- or transpiration-related DEGs were not detected, indicating that differences in fruit maturation cannot be attributed to the expression of photosynthesis- and transpiration-related genes across leaf types.

### 3.2 Foliar CO_2_ and H_2_O gas exchange, WUE and their transcriptomic signatures among citrus hybrids and cultivars

#### 3.2.1 Comparison of foliar gas exchange and WUE among citrus hybrids

**Figure 2:**
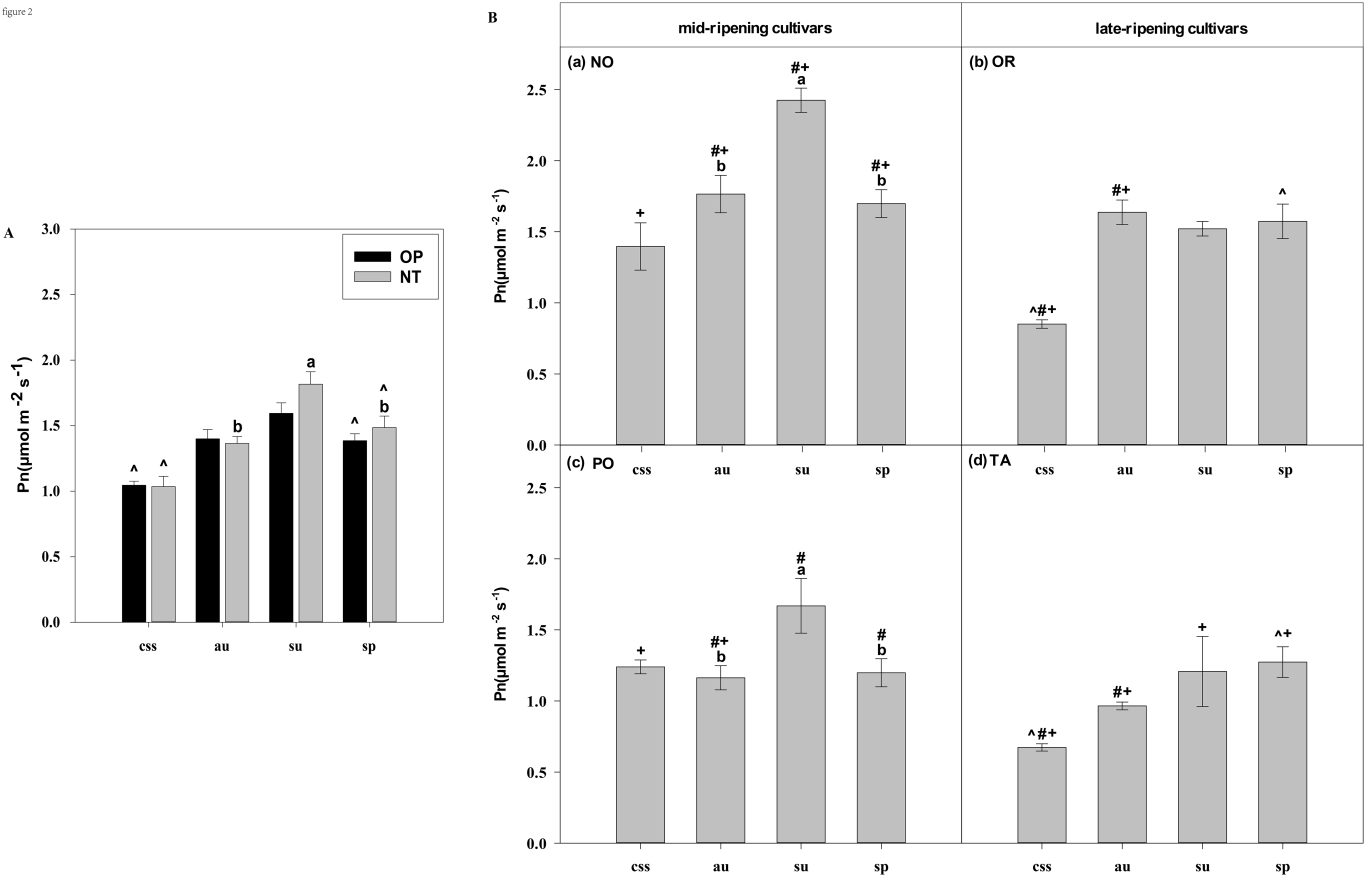
Effects of hybrids, cultivars and leaf types on foliar Pn (net photosynthetic rate). **(A)** represent the Pn of current-year spring shoots (css), autumn (au), summer (su) and spring (sp) of the hybrids OP and NT. **(B)** (a–d) represent the Pn of current-year spring (css), autumn (au), summer (su) and spring shoots (sp) of cultivars Orah (OR), Newhall navel orange (NO), Ponkan (PO), and Tarocco (TA). Data are means (± SE) (n=4). Data are means (± SE) (n=4). Different small letters indicate significant differences between different leaf types; ^ indicates significant differences between different the current-year spring shoots and spring shoots; * indicates significant differences between different the two hybrids (OP and NT); # indicates significant differences between the two hybrids in the same ripening (OR and TA, NO and PO) and + indicates significant differences between the two cultivars (OR and PO, NO and TA) (p < 0.05).

**Figure 3:**
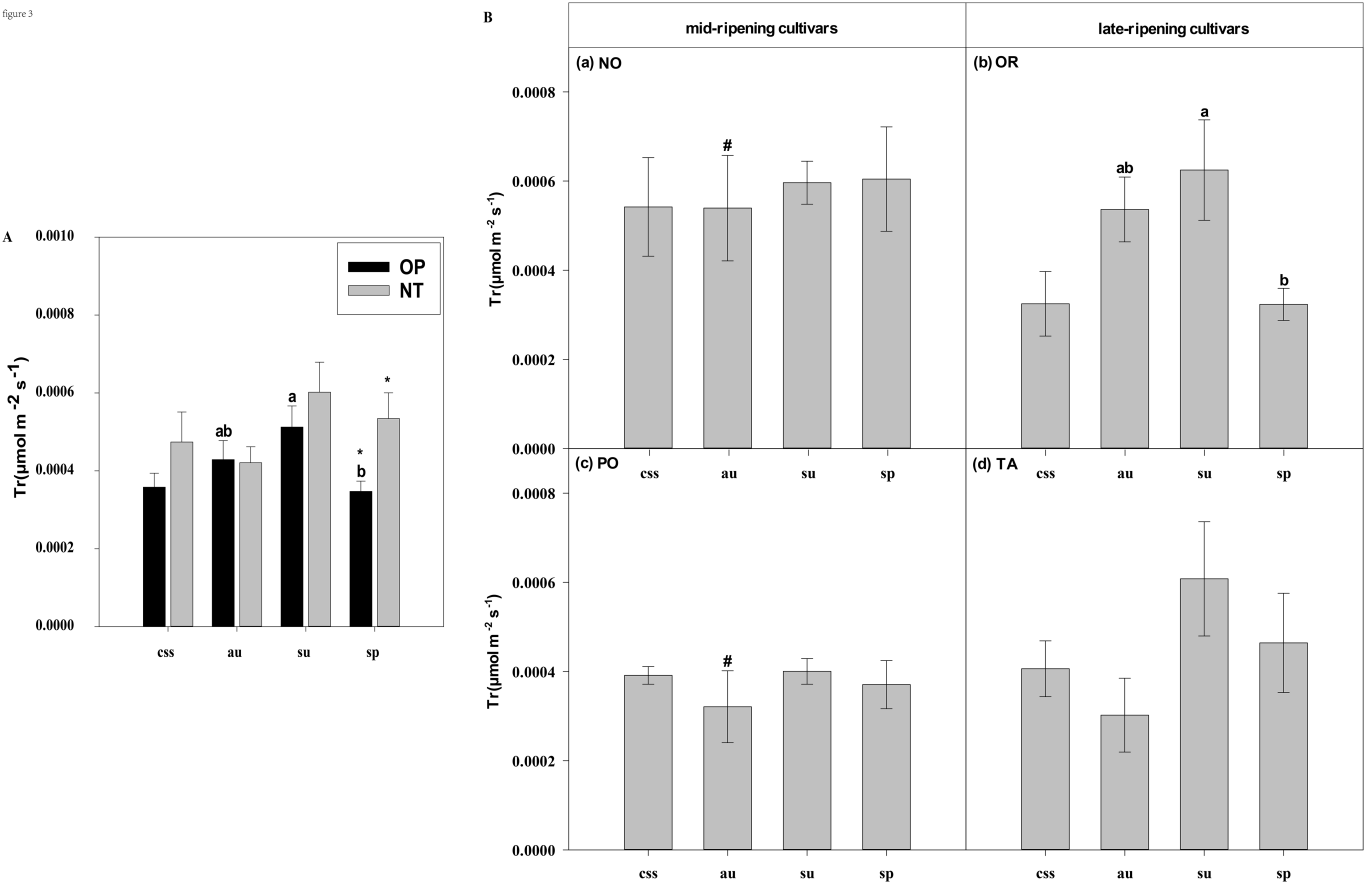
Effects of leaf type on foliar Tr (transpiration rate) in different citrus cultivars. **(A)** represent the Tr of current-year spring shoots (css), autumn (au), summer (su) and spring (sp) of the hybrids OP and NT. **(B)** (a–d) represent the Tr of current-year spring (css), autumn (au), summer (su) and spring shoots (sp) of cultivars Orah (OR), Newhall navel orange (NO), Ponkan (PO), and Tarocco (TA). Data are means (± SE) (n=4). Data are means (± SE) (n=4). Different small letters indicate significant differences between different leaf types; ^ indicates significant differences between different the current-year spring shoots and spring shoots; * indicates significant differences between different the two hybrids (OP and NT); # indicates significant differences between the two hybrids in the same ripening (OR and TA, NO and PO) and + indicates significant differences between the two cultivars (OR and PO, NO and TA) (p < 0.05).

**Figure 4:**
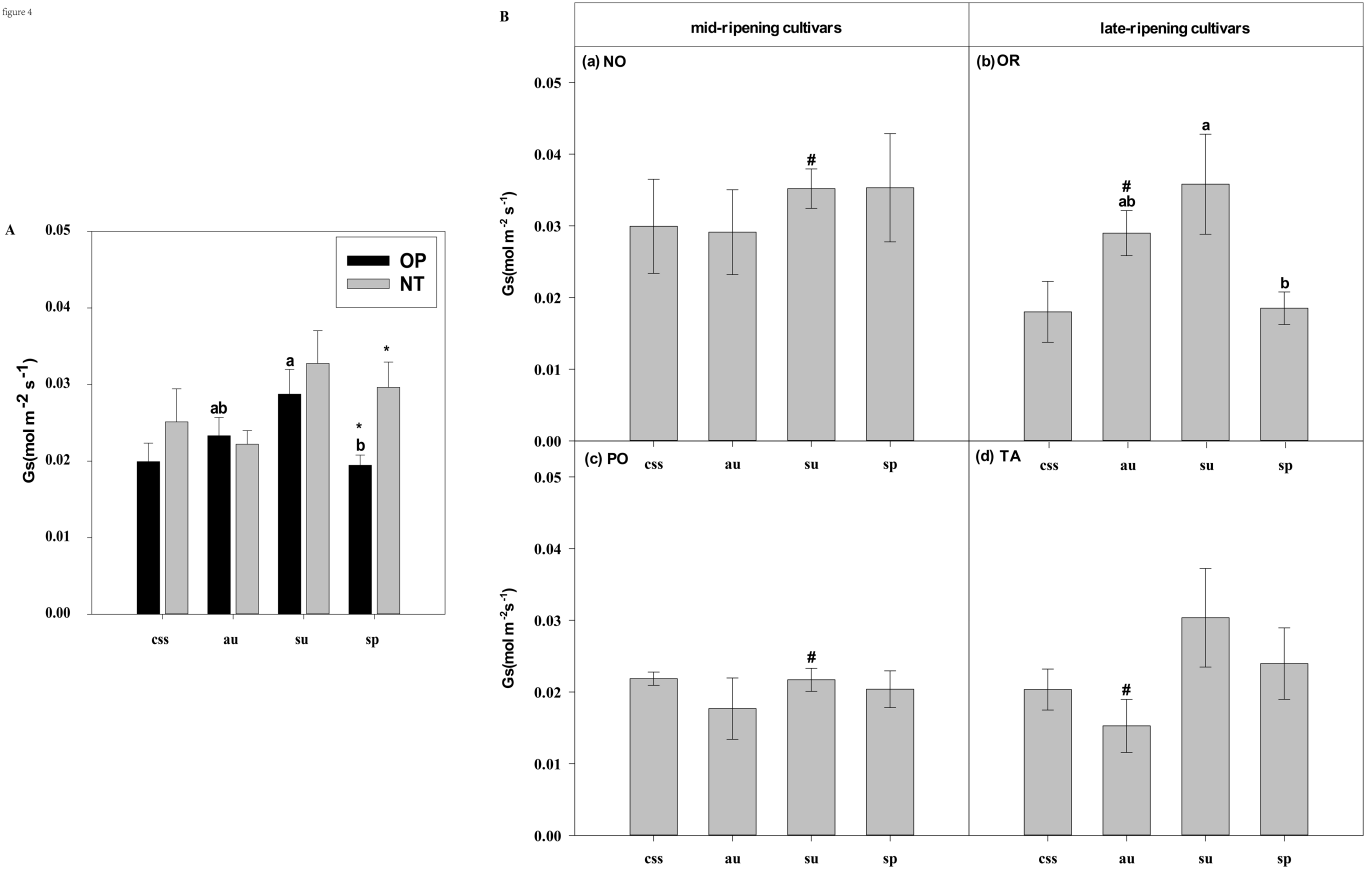
Effects of leaf type on foliar Gs (stomatal conductance) in different citrus cultivars. **(A)** represent the Gs of current-year spring shoots (css), autumn (au), summer (su) and spring (sp) of the hybrids OP and NT. **(B)** (a–d) represent the Gs of current-year spring (css), autumn (au), summer (su) and spring shoots (sp) of cultivars Orah (OR), Newhall navel orange (NO), Ponkan (PO), and Tarocco (TA). Data are means (± SE) (n=4). Data are means (± SE) (n=4). Different small letters indicate significant differences between different leaf types; ^ indicates significant differences between different the current-year spring shoots and spring shoots; * indicates significant differences between different the two hybrids (OP and NT); # indicates significant differences between the two hybrids in the same ripening (OR and TA, NO and PO) and + indicates significant differences between the two cultivars (OR and PO, NO and TA) (p < 0.05).

**Figure 5:**
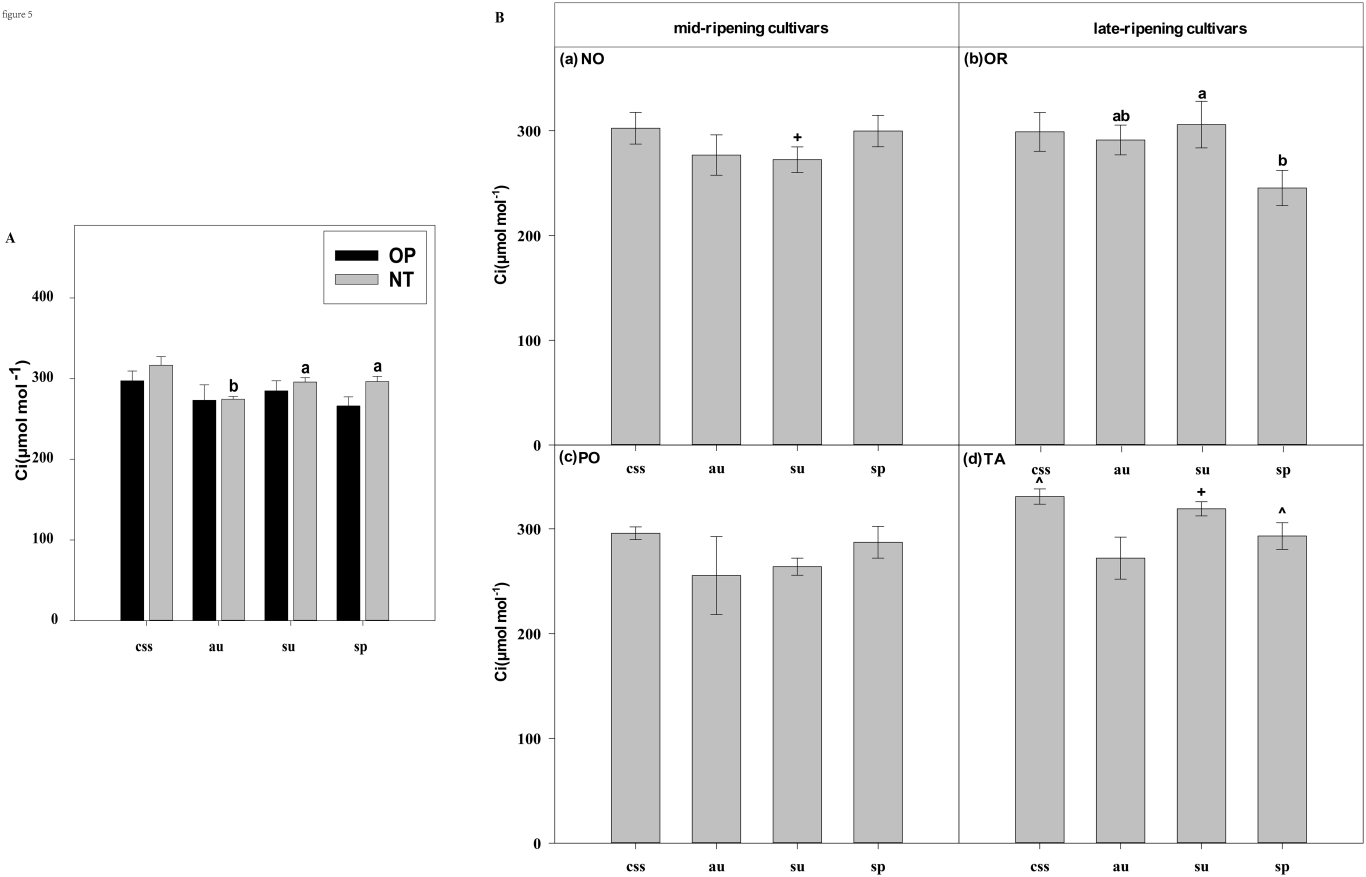
Effects of leaf type on foliar Ci (intercellular CO_2_ concentration) in different citrus cultivars. **(A)** represent the Ci of current-year spring shoots (css), autumn (au), summer (su) and spring (sp) of the hybrids OP and NT. **(B)** (a–d) represent the Ci of current-year spring (css), autumn (au), summer (su) and spring shoots (sp) of cultivars Orah (OR), Newhall navel orange (NO), Ponkan (PO), and Tarocco (TA). Data are means (± SE) (n=4). Data are means (± SE) (n=4). Different small letters indicate significant differences between different leaf types; ^ indicates significant differences between different the current-year spring shoots and spring shoots; * indicates significant differences between different the two hybrids (OP and NT); # indicates significant differences between the two hybrids in the same ripening (OR and TA, NO and PO) and + indicates significant differences between the two cultivars (OR and PO, NO and TA) (p < 0.05).

**Figure 6:**
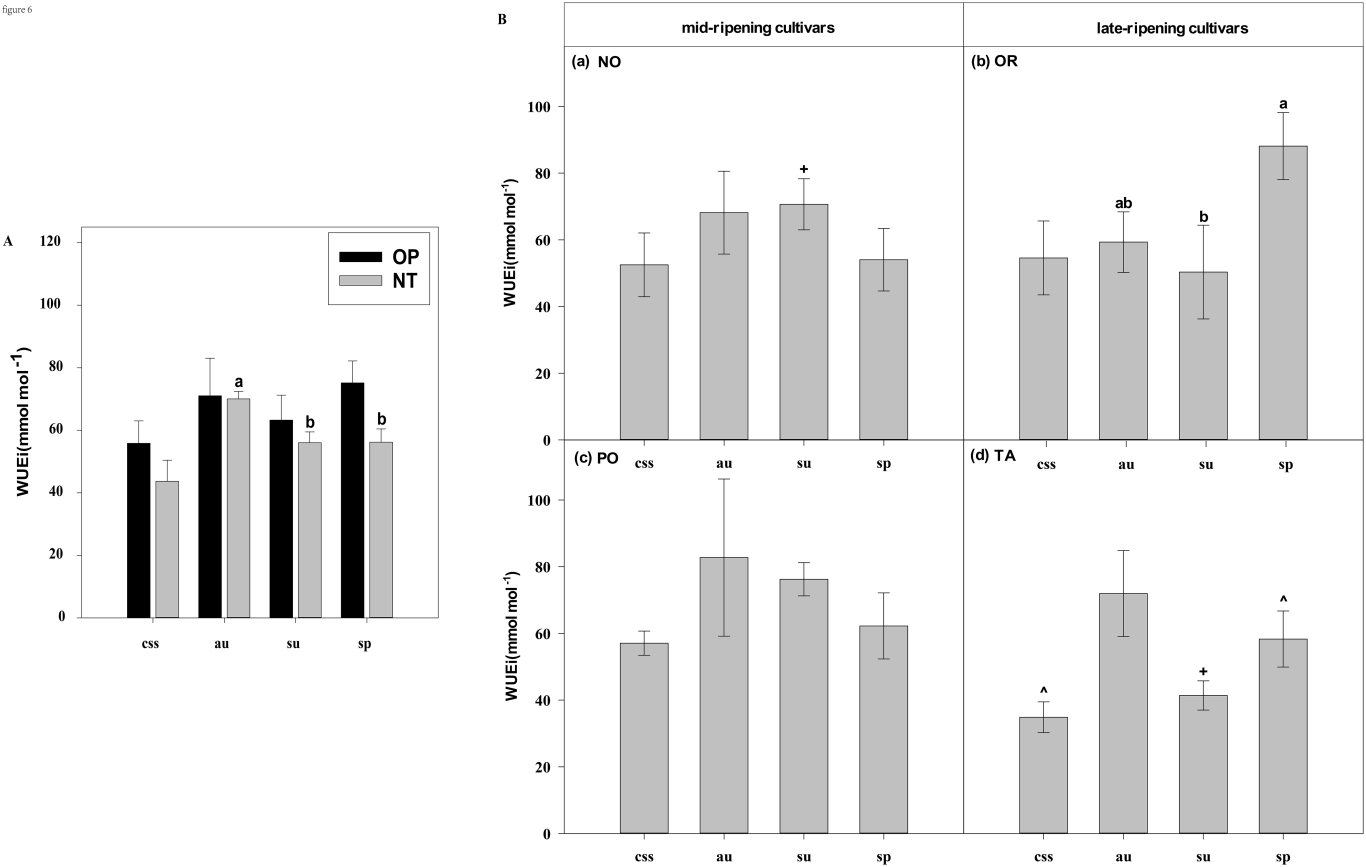
Effects of leaf type on foliar WUEi in different citrus cultivars. **(A)** represent the WUEi of current-year spring shoots (css), autumn (au), summer (su) and spring (sp) of the hybrids OP and NT. **(B)** (a–d) represent the WUEi of current-year spring (css), autumn (au), summer (su) and spring shoots (sp) of cultivars Orah (OR), Newhall navel orange (NO), Ponkan (PO), and Tarocco (TA). Data are means (± SE) (n=4). Data are means (± SE) (n=4). Different small letters indicate significant differences between different leaf types; ^ indicates significant differences between different the current-year spring shoots and spring shoots; * indicates significant differences between different the two hybrids (OP and NT); # indicates significant differences between the two hybrids in the same ripening (OR and TA, NO and PO) and + indicates significant differences between the two cultivars (OR and PO, NO and TA) (p < 0.05).

**Figure 7:**
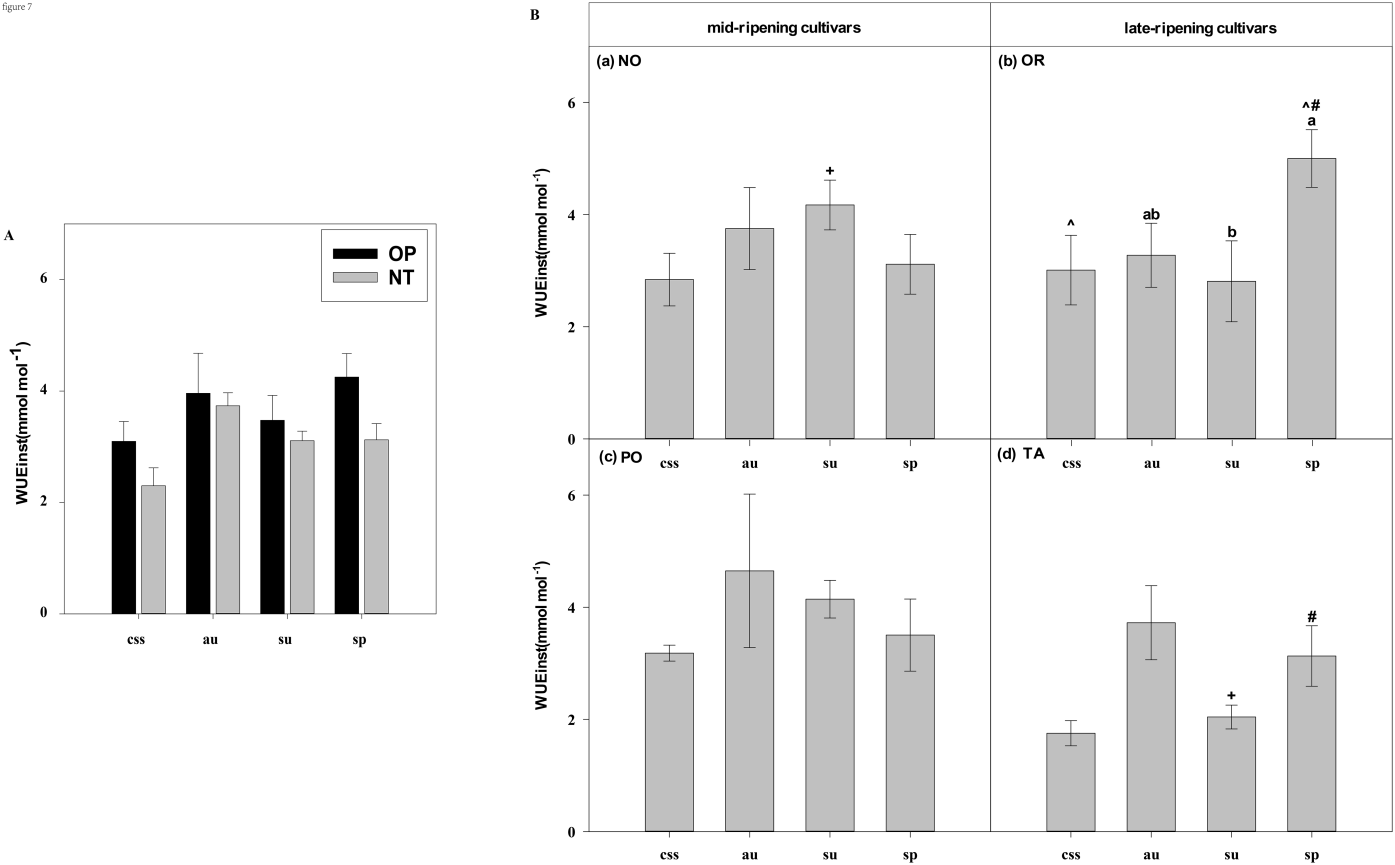
Effects of leaf type on foliar WUEinst in different citrus cultivars. **(A)** represent the WUEinst of current-year spring shoots (css), autumn (au), summer (su) and spring (sp) of the hybrids OP and NT. **(B)** (a–d) represent the WUEinst of current-year spring (css), autumn (au), summer (su) and spring shoots (sp) of cultivars Orah (OR), Newhall navel orange (NO), Ponkan (PO), and Tarocco (TA). Data are means (± SE) (n=4). Data are means (± SE) (n=4). Different small letters indicate significant differences between different leaf types; ^ indicates significant differences between different the current-year spring shoots and spring shoots; * indicates significant differences between different the two hybrids (OP and NT); # indicates significant differences between the two hybrids in the same ripening (OR and TA, NO and PO) and + indicates significant differences between the two cultivars (OR and PO, NO and TA) (p < 0.05).

In current-year spring shoots, the foliar Pn in cultivar OR which belongs to hybrid OP was significantly higher than in cultivar TA which belongs to hybrid NT (**Figure 2B**). Significant differences in foliar Tr, Gs, Ci, WUEi, and WUEinst were not observed between the hybrids (**Figures 3**–**7**). In autumn shoots, the foliar Pn in cultivar NO which belongs to hybrid NT was significantly higher than in cultivar PO which belongs to hybrid OP. Both, foliar Pn and Gs were significantly higher in cultivar OR which belongs to hybrid OP than in cultivar TA which belongs to hybrid NT (**Figures 2B**, **4B**). The foliar Tr in cultivar NO which belongs to hybrid NT was significantly higher than in cultivar PO which belongs to hybrid OP (**Figure 3B**). Differences in Ci, WUEi and WUEinst were not observed between the hybrids across leaf types of autumn shoots (**Figures 5**-**7**). In summer shoots, the foliar Pn and Gs in cultivar NO which belongs to hybrid NT were significantly higher than in cultivar PO which belongs to hybrid OP (**Figures 2B**, **4B**). Differences in Tr, Ci, WUEi and WUEinst were not observed between the hybrids across leaf types of summer shoots (**Figures 3B**, **5**-**7B**). In spring shoots, the foliar Pn in cultivar NO which belongs to hybrid NT was significantly higher than in cultivar PO which belongs to hybrid OP (**Figure 2B**). The foliar Tr and Gs in hybrid NT were significantly higher than in hybrid OP (**Figures 3A**, **4A**). The foliar WUEinst in cultivar OR which belongs to hybrid OP was significantly higher than in cultivar TA which belongs to hybrid NT (**Figure 7B**). Differences in Ci and WUEi were not observed between the hybrids across leaf types of spring shoots (**Figures 5**, **6**). Thus, foliar gas exchange and WUE of citrus leaves were determined by the hybrids.

#### 3.2.2 Comparison of foliar gas exchange and WUE among citrus cultivars

In current-year spring shoots, the foliar Pn of the mid-ripening cultivars NO and PO was significantly higher than in late-ripening cultivars TA and OR of the hybrids NT and OP, respectively (**Figure 2B**). Significant differences in foliar Tr, Gs, Ci, WUEi and WUEinst were not observed between the four cultivars (**Figures 3**-**7**). In autumn shoots, the foliar Pn of late-ripening cultivar OR was significantly higher than in the mid-ripening cultivar PO of hybrid OP, but the foliar Pn of mid-ripening cultivar NO was significantly higher than in the mid-ripening cultivar TA (**Figure 2B**). General differences in Tr, Gs, Ci, WUEi and WUEinst were not observed between mid- and late-ripening cultivars across leaf types on current you spring shoots (**Figures 3**-**7**). In summer shoots, the foliar Pn, WUEi, and WUEinst of mid-ripening cultivar NO was significantly higher compared to the late-ripening cultivar TA (**Figures 2**, **6**, **7**), but the foliar Ci of the mid-ripening cultivar NO was significantly lower than in late-ripening cultivar TA (**Figure 5**). Differences in foliar Tr and Gs were not observed between cultivars across leaf types of summer shoots (**Figures 3**, **4**). In spring shoots, the foliar Pn of mid-ripening cultivar NO was significantly higher compared to late-ripening cultivar TA (**Figures 2A, D**). Differences in foliar Tr, Gs, Ci WUEi, and WUEinst were not observed between the cultivars across leaf types (**Figures 3**-**7**). Thus, foliar gas exchange and WUE of citrus leaves were determined by the cultivars.

#### 3.2.3 Hybrid and cultivar-specific effects of transcriptomic signatures

**Figure 8:**
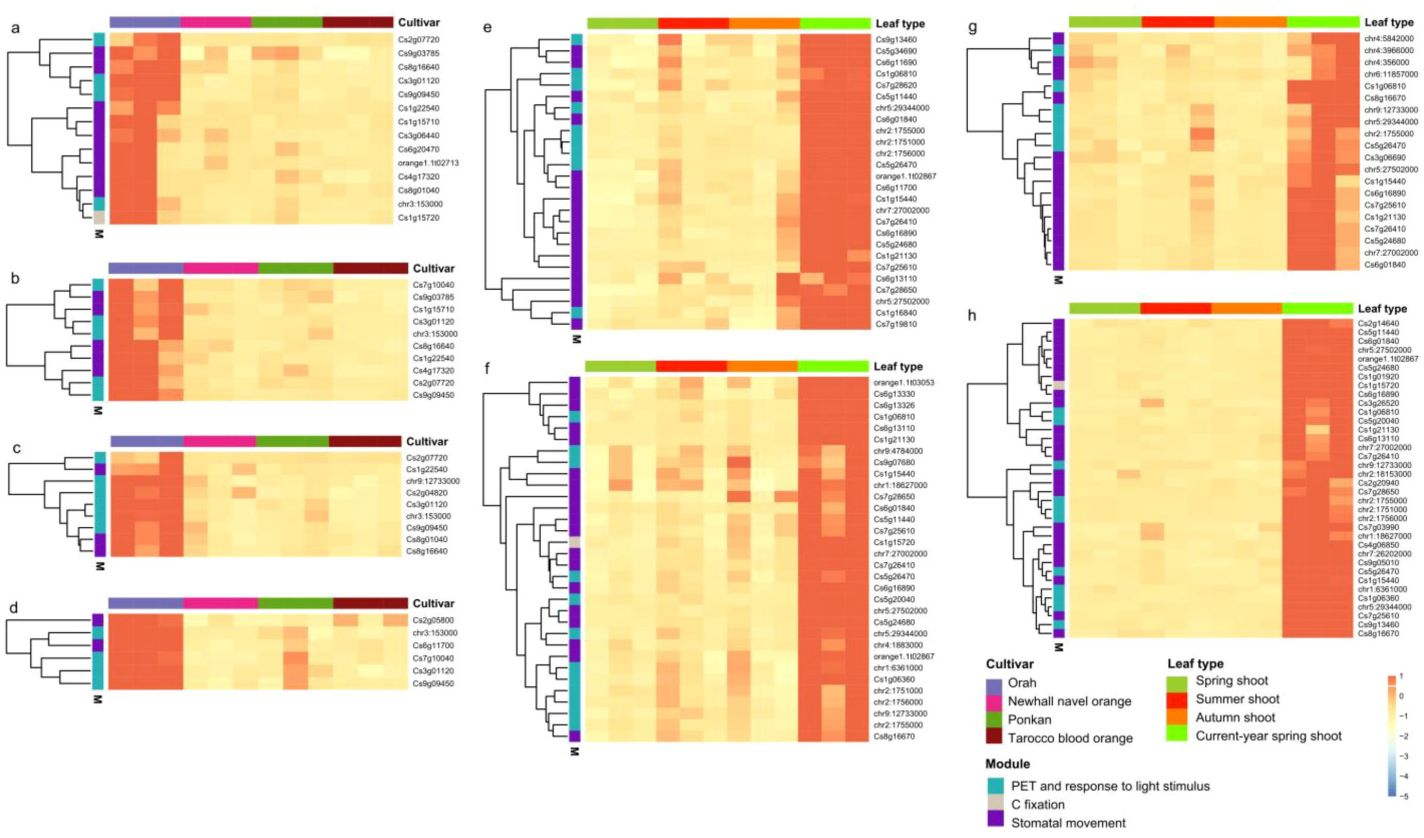
Expression pattern of hybrid/cultivar-specific photosynthesis- and transpiration-related genes across leaf types. Hybrid/cultivar-specific expressed genes in **(A)** spring shoots, **(B)** summer shoots, **(C)** autumn shoots and **(D)** current-year spring shoots. Leaf type-specific expression of genes in **(E)** Orah, **(F)** Newhall navel orange, **(G)** Poken, and **(H)** Tarocco. The expression of genes is centered and scaled in row direction.

Hybrid or cultivar-specific expression of photosynthesis- and transpiration-related genes was studied by transcriptome analysis to reveal differences in the foliar expression profiles. We found that 14, 10, 9 and 6 photosynthesis- and transpiration-related genes were significantly higher expressed in the late-ripening cultivar OR (hybrid OP) in spring, summer, autumn and current-year spring shoots, respectively (**Figures 8A-D**). Three of these genes were shared in the four DEGs sets. These three OR (hybrid OP)-specific highly expressed genes included one responding to light stimuli (Cs3g01120), one involved in photosynthetic electron transport (chr3:153000), and one related to photosystem I (Cs9g09450). The gene set shared by all four cultivars included four photosynthesis-related genes (Cs1g06810, chr5:29344000, chr2:1755000 and Cs5g26470) and eight transpiration-related genes (Cs6g01840, chr7:27002000, Cs7g26410, Cs6g16890, Cs5g24680, Cs1g21130, Cs7g25610 and chr5:27502000). The gene chr2:1755000 encodes a chlorophyll a-b binding protein of the LHCII type 1 functioning as a light receptor to capture and deliver excitation energy to the photosystems. Both chr5:29344000 and Cs5g26470 were GATA transcription factor 5-like genes that specifically bind 5’-GATA-3’ motifs within gene promoters and are probably involved in the regulation of light-responsive genes.

In addition, we identified eight current-year spring shoot-specific, highly up-regulated foliar genes in cultivar PO (hybrid OP) and cultivars NO and TA (hybrid NT), including two encoding chlorophyll a-b binding proteins of LHCII type 1 (chr2:1751000 and chr2:1756000) and the two aquaporin genes TIP2-1 (Cs1g15440) and TIP1-3 (Cs6g13110). Gene chr9:12733000 was annotated as a multiple functional chloroplastic magnesium-chelatase CHLH involved in chlorophyll synthesis, plastid-to-nucleus retrograde signaling and mediating ABA signaling in stomatal guard cells and during seed germination. The chloroplast-localized NADP-dependent malic enzyme (Cs1g15720), a key enzyme of carbon fixation in C4 photosynthesis, was identified in the cultivars NO and TA (hybrid NT). This gene was also highly expressed in the late-ripening cultivar OR (hybrid OP) in spring-shoot leaves. The genes chr1:6361000 and Cs1g06360 shared by the cultivars NO and TA (hybrid NT) are involved in light-harvesting and protein-chromophore linkage. Cs9g07680 and chr9:4784000 identified in mid-ripening cultivar NO (hybrid NT) function as early light-induced protein 1 and take part in the response to cold, heat, and light as well as seed germination and chlorophyll biosynthesis. Cs7g28620 identified in late-ripening cultivar OR (hybrid OP) constitutes a psbP-like protein 1 involved in the assembly of photosystem II super-complexes and required for the adaptation to changing light intensity in photosynthesis (**Figures 8E-H**).

### 3.3 Leaf type-specific effects on foliar CO_2_ and H_2_O gas exchange, WUE and their transcriptomic signatures across citrus hybrids and cultivars

When differences in foliar gas exchange between leaf types were analyzed, in hybrids OP and NT, as well as cultivars OR and TA, the foliar Pn of spring shoots was significantly higher than in current-year spring shoots (**Figures 2A, B**). In hybrid NT and cultivars NO and PO, the foliar Pn of summer shoots was significantly higher than in autumn and spring shoots (**Figures 2A, B**). Only in hybrid OP and the late-ripening cultivar OR, the foliar Tr and Gs of summer shoots were significantly higher than in spring and current-year spring shoots (**Figures 3**, **4**). In the late-ripening cultivar OR, the foliar Ci of summer shoots was significantly higher than in spring shoots. In late-ripening cultivar TA, the foliar Ci of summer and current-year spring shoots were significantly higher than in autumn shoots (**Figures 5B, D**). In late-ripening cultivar OR, the foliar WUEi of spring shoots was significantly higher than in summer shoots (**Figure 6B**). In late-ripening cultivar TA, the foliar WUEi of autumn shoots were significantly higher than in summer and current-year spring shoots (**Figure 6D**). In both, late ripening cultivars FO and TA, the foliar WUEinst of spring shoots were significantly higher than in summer and current-year spring shoots (**Figures 6B, D**). Differences of foliar Ci, WUEi and WUEinst in the mid-ripening cultivars NO and PO between leaf types were not observed (**Figures 5**-**7A, C**). Thus, foliar gas exchange and WUE of citrus leaves were only partially determined by leaf type.

**Figure 9:**
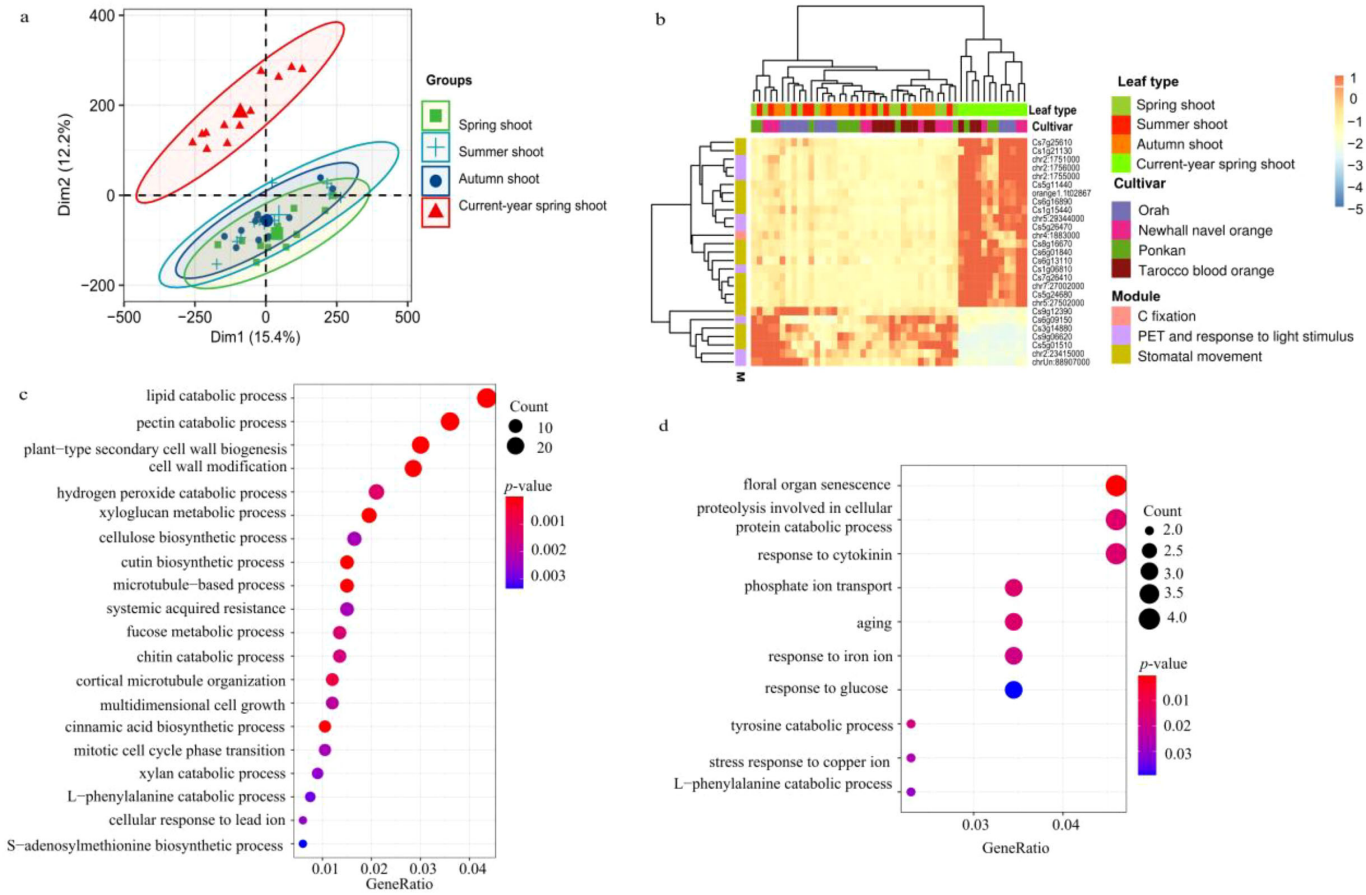
Differentially expressed genes (DEGs) among leaf types. **(A)** Principal component analysis (PCA) revealed the expression variance among leaf types. **(B)** the DEGs between current-year spring shoots and the combination of spring, summer, and autumn shoots; and the expression of genes is centered and scaled in row direction. **(C)** GO and **(D)** KEGG pathway enrichment analysis of significant highly expressed foliar genes.

In order to determine whether leaf types show specific expression profiles of photosynthesis- and transpiration-related genes, we conducted DEGs analysis. However, PCA did not generate two separate clusters in the pairwise comparisons of spring *vs.* summer shoots, spring *vs*. autumn shoots and summer *vs*. autumn shoots. Thus, we detected few to no DEGs in these comparisons. Nevertheless, we found that newly emerging leaves on current-year spring shoots showed a distinct cluster that clearly separated from spring, summer and autumn shoots (**Figure 9A**). These findings suggest that young leaves in spring touch off specific gene expression that would reach a stable level as leaves grow older. Thus, DEGs were detected between current-year spring shoots and the combination of spring, summer and autumn shoots. We identified 198 up-regulated genes in the combination of spring, summer and autumn shoots and 1,091 up-regulated genes in current-year spring shoots. The up-regulated genes in current-year spring shoots were significantly enriched in lipid and pectin catabolic processes, cell wall biogenesis and modification, carbohydrate and xyloglucan metabolism, cellulose biosynthesis, and multidimensional cell growth (**Figure 9C**).

Of these DEGs, three *Lhcb1* genes (chr2:1751000, chr2:1756000 and chr2:1755000) function as light receptor in photosynthetic electron transport and contribute to protein-chromophore linkage and one *rbcS* gene (chr4:1883000) was associated with carbon fixation. Seven DEGs were also identified to be involved in the response to light stimuli, with the COP1-interacting protein 7 (CIP7, Cs1g06810) acting as a light-regulated gene and potentially as a direct target of COP1 for light control of gene expression. Also, gene GATA7 (Cs5g26470) which encodes a transcription factor, probably involved in the regulation of light-responsive genes, showed significant differential expression between current-year spring shoots and leaf types. The DEGs further included 13 genes that are thought to take part in the response to water deprivation (orange1.1t02867, chr7:27002000, chr5:27502000, Cs7g26410, Cs1g21130, Cs6g01840 and Cs5g24680), the regulation of stomatal aperture (Cs5g11440, Cs6g16890, Cs6g13110 and Cs8g16670), and water transport (Cs1g15440 and Cs7g25610) (**Figure 9B**).

The genes identified as important regulators involved in the response to water deprivation and stomatal aperture overlapped with those found between current-year spring shoots and in the combination of spring, summer, and autumn shoots. The 198 highly expressed genes in the combination of spring, summer and autumn shoots compared with current-year spring shoots were significantly enriched in genes involved in floral organ senescence, cellular protein catabolic processes, cytokinin regulation, phosphate ion transport and aging (**Figure 9D**). Only seven photosynthesis- and transpiration-related genes were identified in this gene set, including four activated in response to water deprivation (Cs3g14880, Cs9g06620, Cs5g01510 and Cs9g12390), one ferritin-encoded gene (Cs6g09150) thought to function as an iron source for the synthesis of iron-containing proteins involved in photosynthesis at early stages of development, one gene (chrUn:88907000) of the oxidative photosynthetic carbon pathway, and one (chr2:23415000) functioning in chloroplast organization and responding to light stimuli (**Figure 9B**).

We also found three foliar genes (Cs2g07720, Cs8g16640 and Cs1g22540) shared by spring, summer and autumn shoots and one (Cs8g01040) specifically expressed in spring, autumn and current-year spring shoots (**Figures 8A-D**). Particularly, Cs2g07720 is a multifunctional gene responsible for the transport of phosphoglycerate, phosphoenolpyruvate, and glucose-6-phosphate in the regulation of photosynthesis. Cs8g16640 encodes aquaporin PIP2–1 facilitating water transport across cell membranes, while Cs1g22540 encodes a mitogen-activated protein kinase 18 (MAPKKK18) reported to enhance plant drought resistance by accelerating stomatal closure. Cs8g01040 encodes a phototropin-2-related gene controlling a range of plant responses including chloroplast relocation, stomatal opening, and phototropism. Our results also revealed highly up-regulated genes in current-year spring shoots of the four citrus cultivars studied (**Figures 8E-H**).

To sum up, the differences in gas exchange only partially match differences in gene expression, therefore differences in gas exchange are more related to posttranscriptional rather than transcriptional regulation.

## 4 Discussion

Photosynthesis and WUE of plants are mainly influenced by plant morphology, growth and developmental stages (Ribeiro et al., 2012; Xiao et al., 2014; Xiong et al., 2020; Munjonji et al., 2021). Although previous studies have investigated the photosynthetic characteristics of citrus, to our best knowledge, this is the first study analyzing, to which extent differences in photosynthesis and water use between hybrids, cultivars and leaf types of citrus are regulated by changes in the expression of photosynthesis- and transpiration-related genes.

### 4.1 Characterization of foliar photosynthesis and WUE traits among citrus hybrids and their transcriptomic signatures

Our results show that the characteristics of photosynthesis and WUE traits in citrus leaves are influenced by the different hybrids investigated, in agreement with hypothesis (i) (**Figures 1**-**7**). In summer shoots, the foliar Pn and Gs in cultivar NO which belongs to hybrid NT were significantly higher than in cultivar PO which belongs to hybrid OP (**Figures 2B**, **4B**). In spring shoots, the foliar Pn in cultivar NO which belongs to hybrid NT was significantly higher than in cultivar PO which belongs to hybrid OP (**Figure 2B**) and the foliar Tr and Gs in hybrid NT were significantly higher than in hybrid OP (**Figures 3A**, **4A**). This result is similar to research on citrus under drought stress: under drought and control conditions, compared with hybrid OP (OR), hybrid NT(NO) exhibited better physiological performance (increased Pn in leaves) (Jia et al., 2024). However, the foliar WUEinst in cultivar OR which belongs to hybrid OP was significantly higher than in cultivar TA which belongs to hybrid NT (**Figure 7B**). This is to be expected, because enhanced stomatal conductance and transpiration rates lead to increased photosynthesis, which also leads to reduced water use efficiency. In this context, instantaneous water use efficiency is of stronger ecological or agricultural relevance than intrinsic water use efficiency, since it is directly related to water consumption by transpiration rather than stomatal conductance.

In the present study, expression of foliar photosynthesis- and transpiration-related genes of mid-ripening (i.e., NO and PO) and late-ripening (i.e., OR and TA) citrus cultivars were compared across investigated leaf types. However, photosynthesis- or transpiration-related DEGs were not detected indicating that differences in fruit maturation cannot be attributed to the expression of photosynthesis- and transpiration-related genes, because only two genes (Cs1g12660 and Cs7g03150) were shared between these DEG sets. These genes have functions not directly related to fruit maturation, *i.e.* Cs1g12660 is supposed to take part in the methylation pathway of lignin biosynthesis, while Cs7g03150 constitutes a multifunctional gene involved in the responses to abiotic stress such as salt, cold, and heat as well as biotic stress via regulation of abscisic acid biosynthesis. However, we found that such genes were significantly higher expressed in the hybrid OP (cultivar OR) in spring, summer, autumn and current-year spring shoots, demonstrating differences in gene expression of photosynthesis and transpiration between citrus hybrids OP and NT (**Figures 8A-D**). Similar results have been reported in other studies, such as, in corn hybrids, where changes in the transient binding activity of the circadian clock gene Zm CCA1 increased the expression level of genes related to photosynthetic carbon fixation, which enhances the yield in the hybrids compared to their parents (Ko et al., 2016).

### 4.2 Characterization of foliar photosynthesis and WUE traits among citrus cultivars and their transcriptomic signatures

Our results show that the characteristics of photosynthesis and WUE traits in citrus leaves are influenced by the cultivars investigated, in agreement with hypothesis (i) (**Figures 1**-**7**). In spring, summer, autumn and current-year spring shoots, foliar Pn of the mid-ripening cultivar NO was significantly higher than the late-ripening cultivar TA (**Figure 2B**). It is consistent with a previous study on zinc-rich soil indicating that the photosynthesis of these citrus cultivars is significantly different, with the foliar Pn of cultivar NO being significantly higher than that of cultivar TA (Chen et al., 2014). This observation can be attributed to differences in leaf morphology between the cultivars. Also, research on tea trees showed a large diversity in leaf morphology and physiological activities among different cultivars (Feng et al., 2014). The foliar Ci of the mid-ripening cultivar NO was significantly lower than in late-ripening cultivar TA in summer shoots, but the foliar WUEi and WUEinst of the mid-ripening cultivar NO was significantly higher than in the late-ripening cultivar TA in the summer shoots (**Figures 5**-**7**). Previous studies on apple also indicated large differences in photosynthesis and WUE across cultivars, *e.g.*, the cultivar ‘Braeburn’ being more conservative in WUE than the cultivar ‘Fuji’, due to stomatal limitation of CO_2_ assimilation and higher WUEi (Massonnet et al., 2007). Apparently, the foliar Ci is increased in cultivars of these fruit tree species to improve WUE.

Genes related to photosynthesis and transpiration were significantly higher expressed in the late-ripening OR cultivar in spring, summer, autumn and current-year spring shoots, demonstrating differences between citrus cultivars OR and PO of hybrid OP (**Figures 8A-D**). Studies on cotton also showed that, compared to conventional cultivars, the Bt cultivar exhibits lower stomatal conductance, net photosynthetic and transpiration, but higher instantaneous and long-term water use efficiency (Guo et al., 2016). Also in citrus, decreased foliar Gs was found in all cultivars under water deficit, which was associated with reduced PIP2.1 expression (Miranda et al., 2022). If cultivars OR and PO of hybrid OP analyzed in the present study also differ in the response to water deficit remains to be elucidated.

### 4.3 Effects of leaf type on photosynthesis and WUE traits of citrus leaves, and their transcriptomic signatures

Our results showed that the characteristics of photosynthesis and WUE in citrus leaves partially depended on leaf type in agreement with hypothesis (i) (**Figures 2**-**7**). In late-ripening cultivars OR and TA of hybrids OP and NT, the foliar Pn of spring shoots was significantly higher than in current-year spring shoots (**Figures 2A, B**). This finding can be attributed to the difference in maturity of spring shoot leaves compared to current-year spring shoot leaves (hypothesis ii). The current-year spring shoot leaves are in an early development stage with weak photosynthetic activity. However, in mid-ripening cultivars NO and PO, the foliar Pn of summer shoots was significantly higher than in autumn and spring shoots (**Figure 2B**), because the maturity of spring and summer shoot leaves was relatively high, but the photosynthetic activity of spring shoot leaves began to decline and senesce. Also, previous studies with annual, deciduous and evergreen plants showed that leaf age strongly affect the photosynthetic capacity (Mooney and Gulmon, 1982) that was lower in young compared to mature leaves (Shirke, 2001). However, the foliar Pn of leaves decreased with the increasing leaf age in Mediterranean oak (Alonso-Forn et al., 2022). Similarly, in evergreen citrus Huangguogan and *Salustiana* sweet orange trees, the foliar Pn of summer shoot leaves was significantly higher than in spring shoot leaves (Xiong et al., 2020; Nebauer et al., 2013). Apparently, the progressing maturity of spring shoot leaves significantly reduced their physiological activity compared to younger summer shoot leaves.

Foliar Gs, Tr, and WUE of evergreen tree species leaves are also closely related to leaf age (Chondrogiannis and Grammatikopoulos, 2016; Albert et al., 2017). In this study, the foliar Gs and Tr of the younger summer shoot leaves of hybrid OP and cultivar OR were significantly higher than in the older spring shoot leaves (**Figures 3**, **4**), whereas foliar WUEi and WUEinst of spring shoot leaves were significantly higher in summer shoot leaves in cultivar OR (**Figures 6**, **7**). Apparently, leaf age affected foliar Gs, Tr and, subsequently WUE in OR cultivar. Also, previous field and laboratory studies with other evergreen trees, *e.g., Cyclobalanopsis glauca* seedlings, indicated that Gs in young leaves is significantly higher than in mature leaves. In evergreen *Capparis aristiguetae* Iltis and *Morisonia americana* L Tr was significantly higher in young than in old leaves, whereas WUE of old leaves was significantly higher than in young leaves (Sobrado, 1994; Zhang et al., 2014).

The Pn of evergreen trees is usually relatively low compared to deciduous trees because of high mesophyll resistance (Loreto et al., 1992). Low Pn was observed for citrus in previous studies too (Qi et al., 2021; Santos et al., 2011). In the present study, the Pn of current-year spring shoots of citrus was even lower than reported for spring shoots of Citrus cultivar “HuangGuogan” by Xiong et al. (2020). This difference can be attributed to the relatively dry climate in the year of the present study, supporting the view that evergreen species possess lower photosynthesis and transpiration rates during dry periods to conserve water (Tomlinson et al., 2013). In addition, the low spring temperatures at the location of the present study may have affected net photosynthesis of current-year spring shoots of the citrus trees (Primo-Capella et al., 2021).

Differences in photosynthesis and foliar WUE between leaf types are assumed to be regulated by changes in the expression of photosynthesis and transpiration-related genes (Lin M. et al., 2015; Ding et al., 2016; Zhang et al., 2018; Ribeiro et al., 2021). In our results as hypothesized (iii), genes involved in lipid and pectin catabolic processes, cell wall biogenesis and modification, carbohydrate and xyloglucan metabolic processes, cellulose biosynthesis, and multidimensional cell growth were significantly up-regulated in current-year spring shoot leaves (**Figures 9B**). This result is not surprising, since current-year spring shoots are in the period of vigorous growth, cell wall generation, and accelerated carbohydrate and cellulose synthesis that all require high rates of photosynthesis as source input. Similarly, carbohydrate biosynthesis, photosynthesis, starch biosynthesis, and disaccharide metabolic processes were found to be enriched among the up-regulated differentially expressed genes (DEGs) in mature compared to young leaves of citrus (Ribeiro et al., 2021). In this study, highly expressed genes were involved in floral organ senescence, cellular protein catabolic processes, cytokinin regulation, phosphate ion transport and aging, when combined data of spring, summer and autumn shoots were compared with the current-year spring shoots (**Figure 9D**). This finding indicates that during leaf development the expression of senescence-related genes was also gradually enhanced. These results on citrus leaves are consistent with the observation on senescing cotton leaves, showing down-regulation of most genes related to photosynthesis, chlorophyll metabolism and carbon fixation during leaf maturation (Lin M. et al., 2015). Also in other species, *e.g.*, cassava (*Manihot esculenta* Crantz), genes related to cell wall synthesis and basic cellular metabolism were reported to be highly expressed in young developing leaves, whereas genes involved in lipid metabolism and tetrapyrrole synthesis were highly expressed at the transition to maturation, and genes related to photosynthesis and carbohydrate metabolism were highly expressed in mature leaves (Ding et al., 2016). Similarly, in the subtropical forest tree species *Schima superba* and *Cryptocarya concinna*, compared young leaves with the mature leaves, photosynthesis-related genes were extensively downregulated and flavonoid-pathway-related genes were extensively upregulated (Zhang et al., 2018). Therefore, in citrus plants, metabolic processes are largely regulated by leaf development at the level of gene expression, as previously reported for other species as in line with our hypothesis (iii).

The photosynthetic performance of citrus leaves is also affected by seasonal changes which are related to the seasonal development of citrus leaves. For example, Ribeiro et al. (2009a, b; 2012) reported that citrus leaves’ photosynthesis was significantly higher in summer than in winter. Our study indicates that the expression of related genes is affected by leaf type and cultivar (hypothesis i and iii). Similarly, in evergreen conifers, seasonal changes can be related to differences in gene expression. Bag et al. (2021) reported that the seasonal changes in the transcriptome profiles of major gene families were related to needle development in the evergreen conifer of Norway spruce. However, some studies showed that gene expression could be affected by leaf types (young and old leaves) more than cultivar and seasonal variation (*e.g.*, Kumar et al., 2016). In contrary, studies of citrus photosynthesis indicated that seasonal changes of Gs, Pn, Tr, and WUE were more pronounced than variations between cultivars (*e.g.*, Munjonji et al., 2021). Therefore, whether seasonal changes of photosynthesis in citrus leaves are controlled at the physiological and/or molecular levels still remains to be elucidated.

## 5 Conclusion

We observed differences in CO_2_ and H_2_O gas exchange, WUE and the expression of genes involved in photosynthesis and transpiration between different leaf types of two hybrids in four citrus cultivars differing in fruit maturation. The photosynthesis and WUE are highly dependent on hybrid, cultivar and leaf type and these differences can partially be explained by differences in expression of photosynthesis- and transpiration-related genes. Genes involved in lipid and pectin catabolic processes, cell wall biogenesis and modification, carbohydrate and xyloglucan metabolism, cellulose biosynthesis and cell growth were significantly upregulated in current-year spring shoots compared to the other leaf types investigated. Expression of photosynthesis- and transpiration-related genes was significantly enhanced in leaves of late-ripening cultivar OR compared to the other cultivars. However, the differences between hybrids and cultivars observed could not be attributed to differences in fruit maturation. The results of these snapshot measurements can be affected by many environmental and climate history effects that were not analyzed in the present study. Based on the results of the present transcriptome analyses, still attractive candidate genes can be identified for future selection of citrus trees with efficient photosynthetic capacity and economic water use efficiency.

## Data Availability

The original contributions presented in the study are included in the article/**Supplementary Material**. Further inquiries can be directed to the corresponding author.

## Supplementary material

Supplementary Figure 1 Partial least squares-discriminant analysis (PLS-DA) scores of gas exchange parameters of different hybrids in each leaf type and different leaf types in each hybrid. **(A–D)** show the differences between the two hybrids including OP and NT for each leaf type including current-year spring (css), autumn (au), summer (su), and spring (sp) shoots. **(E, F)** show the differences between the leaf types for the two hybrids OP and NT. The PLS-DA loading charts contain the parameters Pn (net photosynthesis), Tr (transpiration), Gs (stomatal conductance), Ci (intracellular CO_2_ concentration), WUEi (intrinsic water use efficiency), and WUEinst (instantaneous water use efficiency). The ellipses indicate the 95% confidence range.

Supplementary Figure 2 Expression pattern of photosynthesis- and transpiration- related genes. Expression pattern of **(A)** photosynthesis electron transport-involved genes and **(B)** participated in the carbon fixation via Calvin-Benson cycle genes and **(C)** control leaf water status via regulation of stomatal movement. The expression of genes is centered and scaled in row direction.

Supplementary Tables 1-4 The Tables S1–4 represent DEGs between mid-ripening and late-ripening cultivars at spring, summer, autumn and current-year spring shoots respectively.
