# Supplementary figures and images for: Efficient photosynthesis and economic water use of citrus leaves depend on hybrid, cultivar and leaf type
Source: Front Plant Sci. 2025 Sep 4;16:1536703. doi: 10.3389/fpls.2025.1536703 (PMC12443699; doi:10.3389/fpls.2025.1536703)

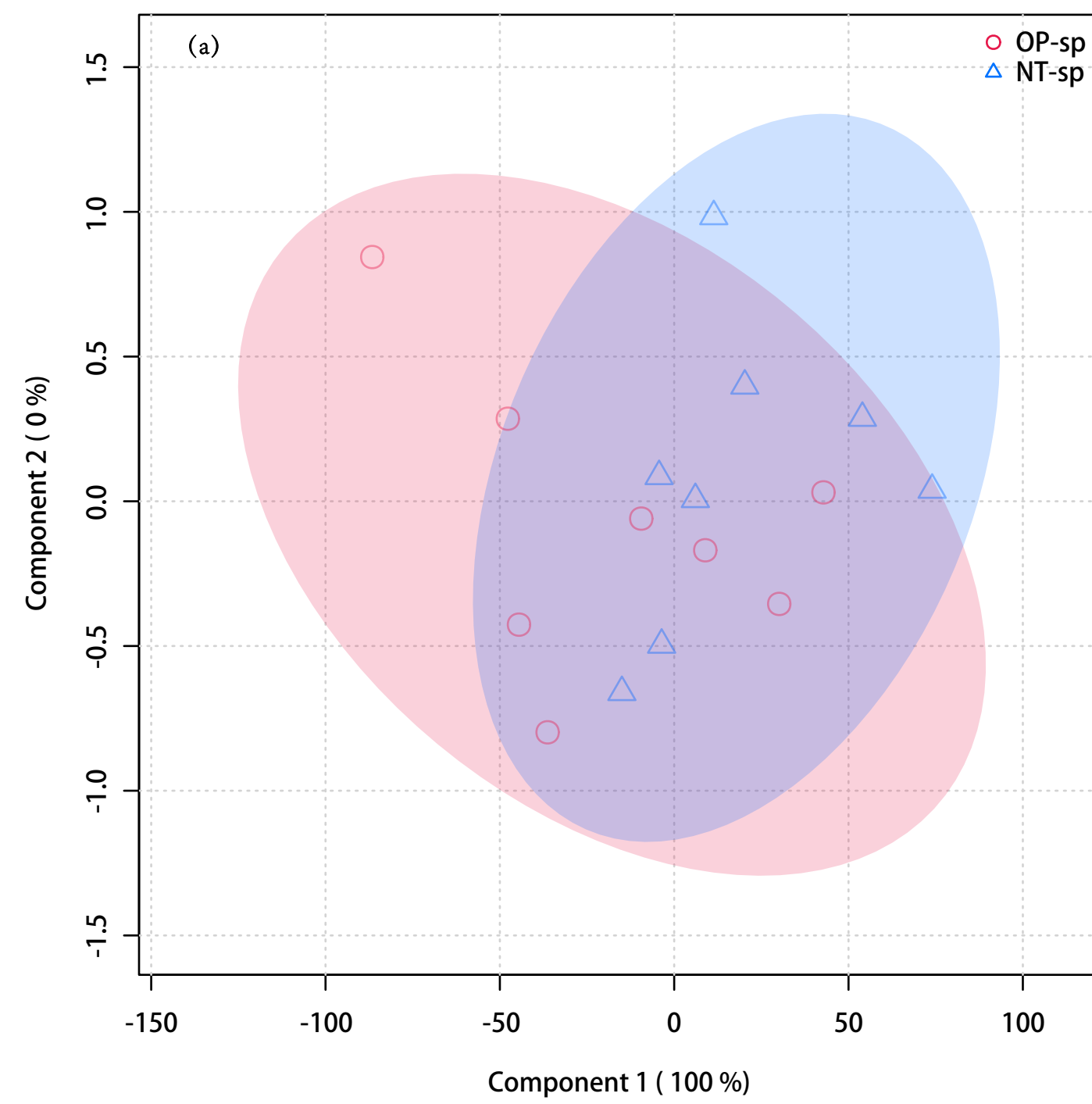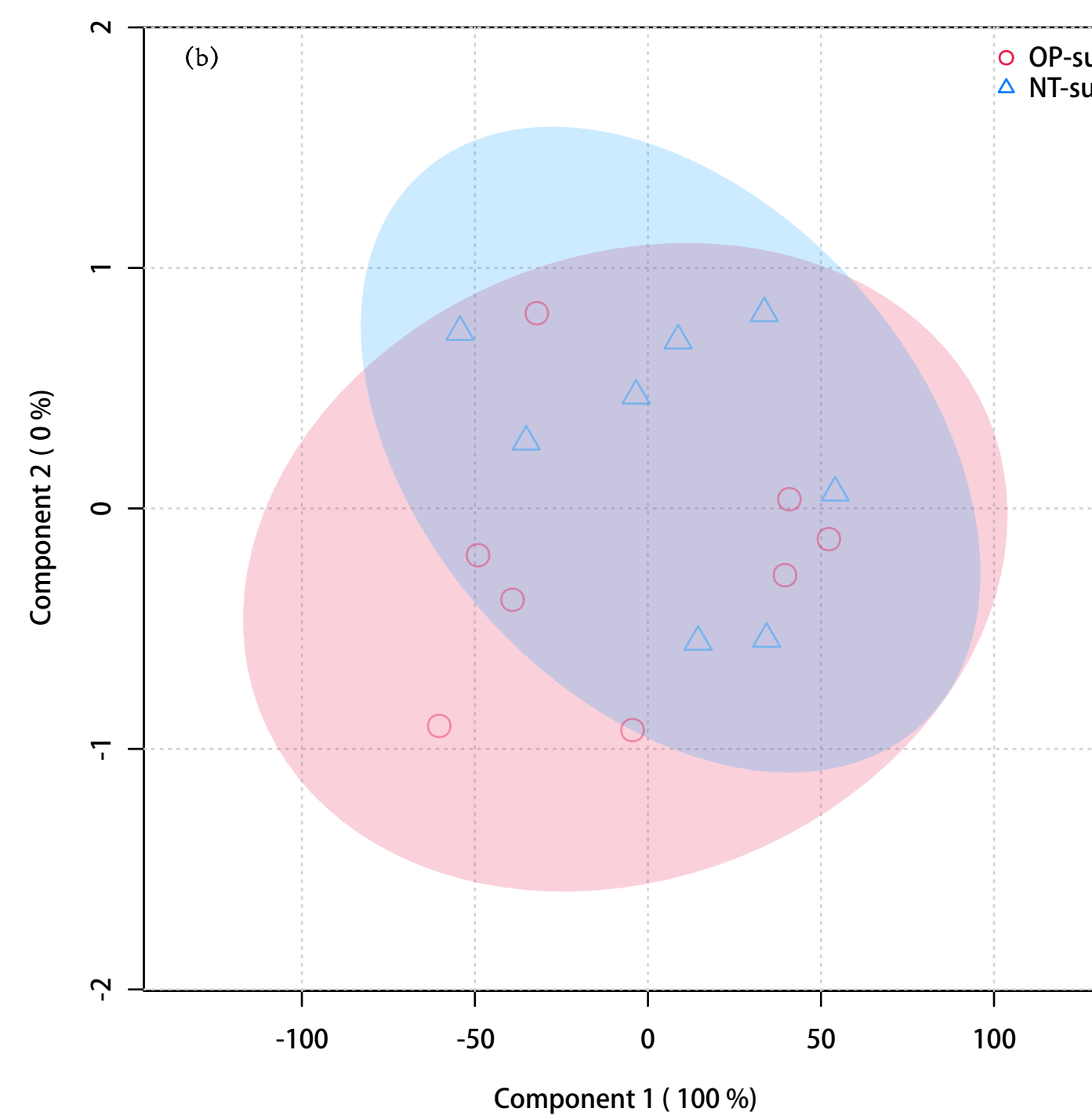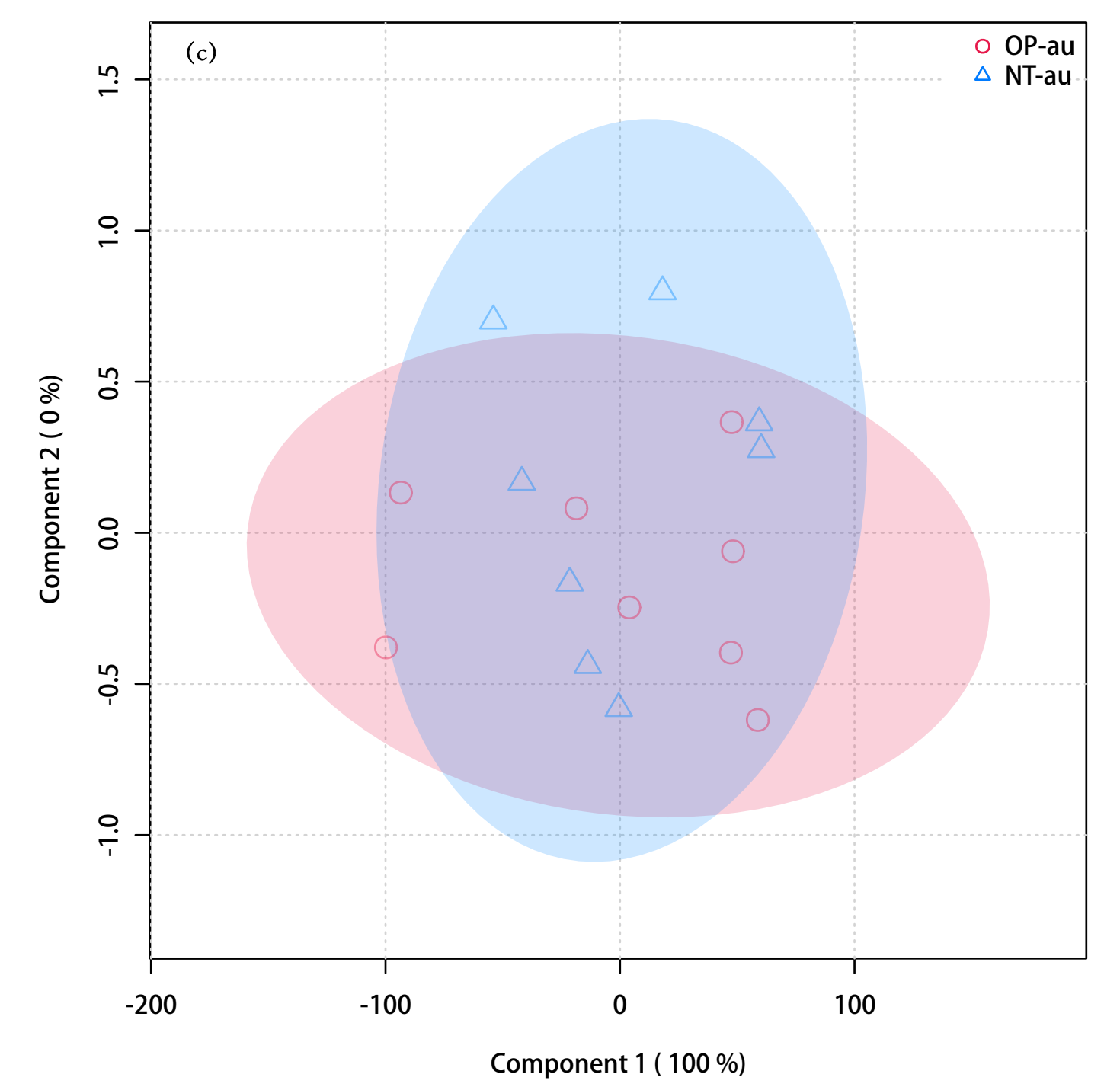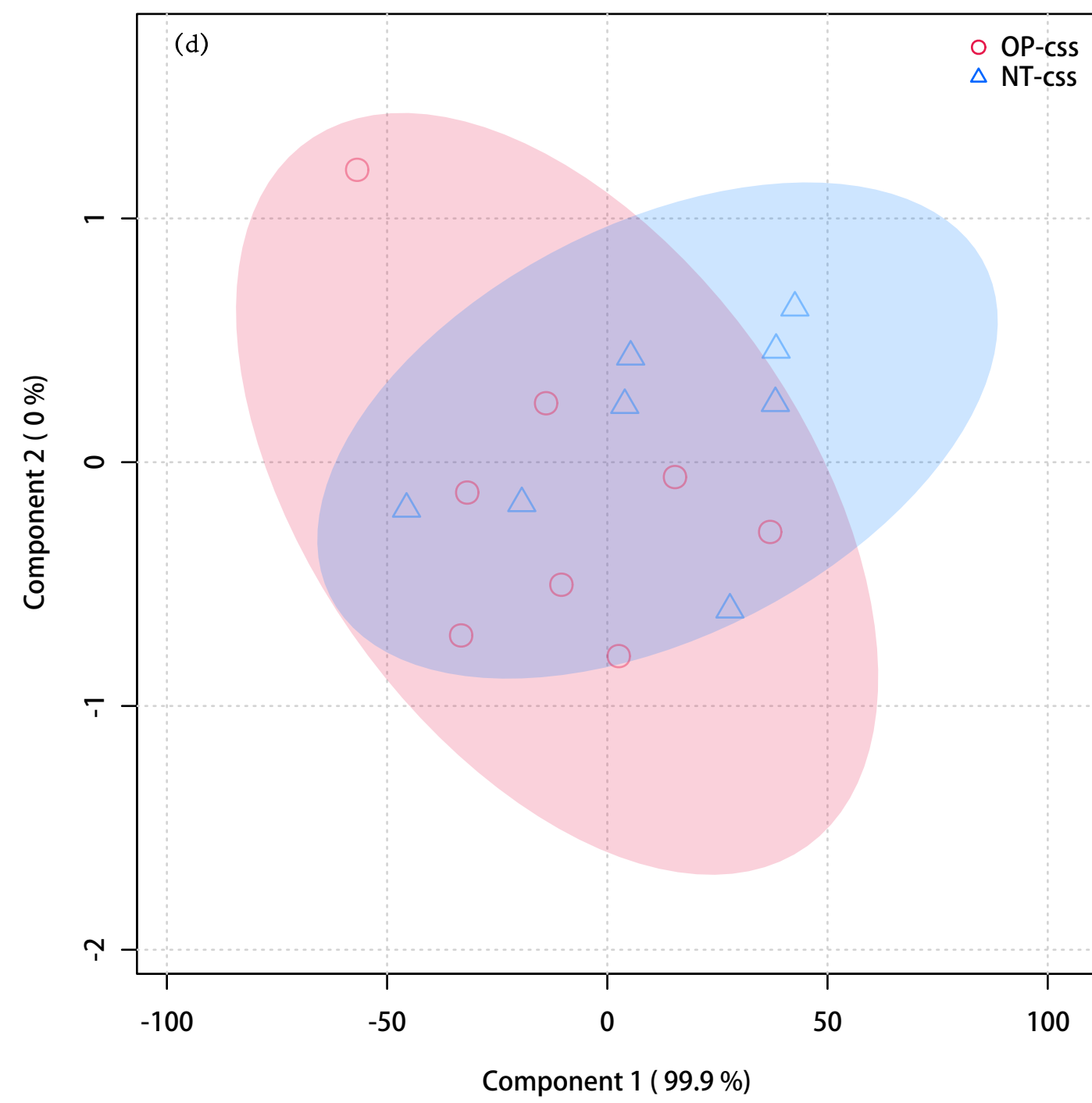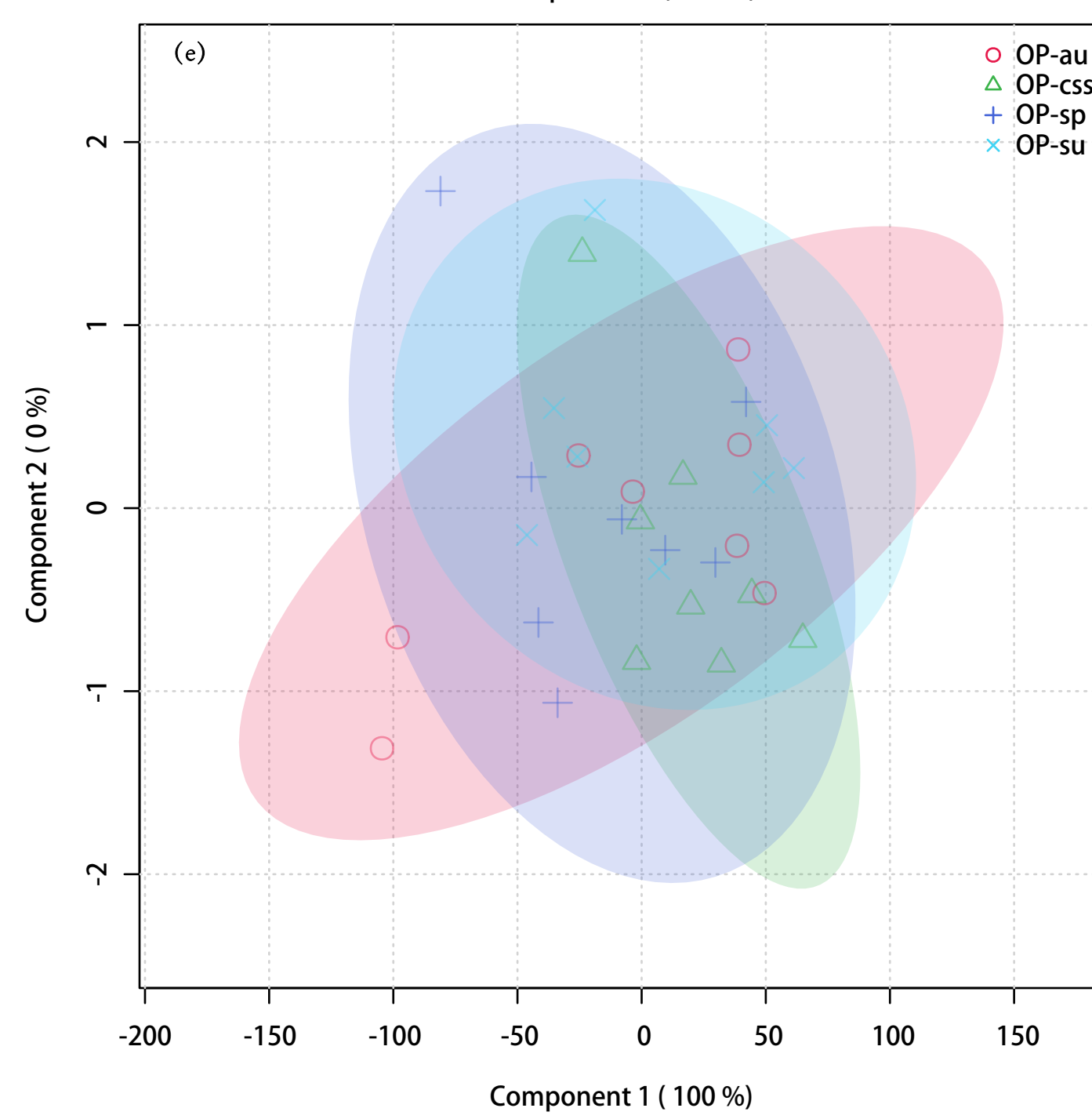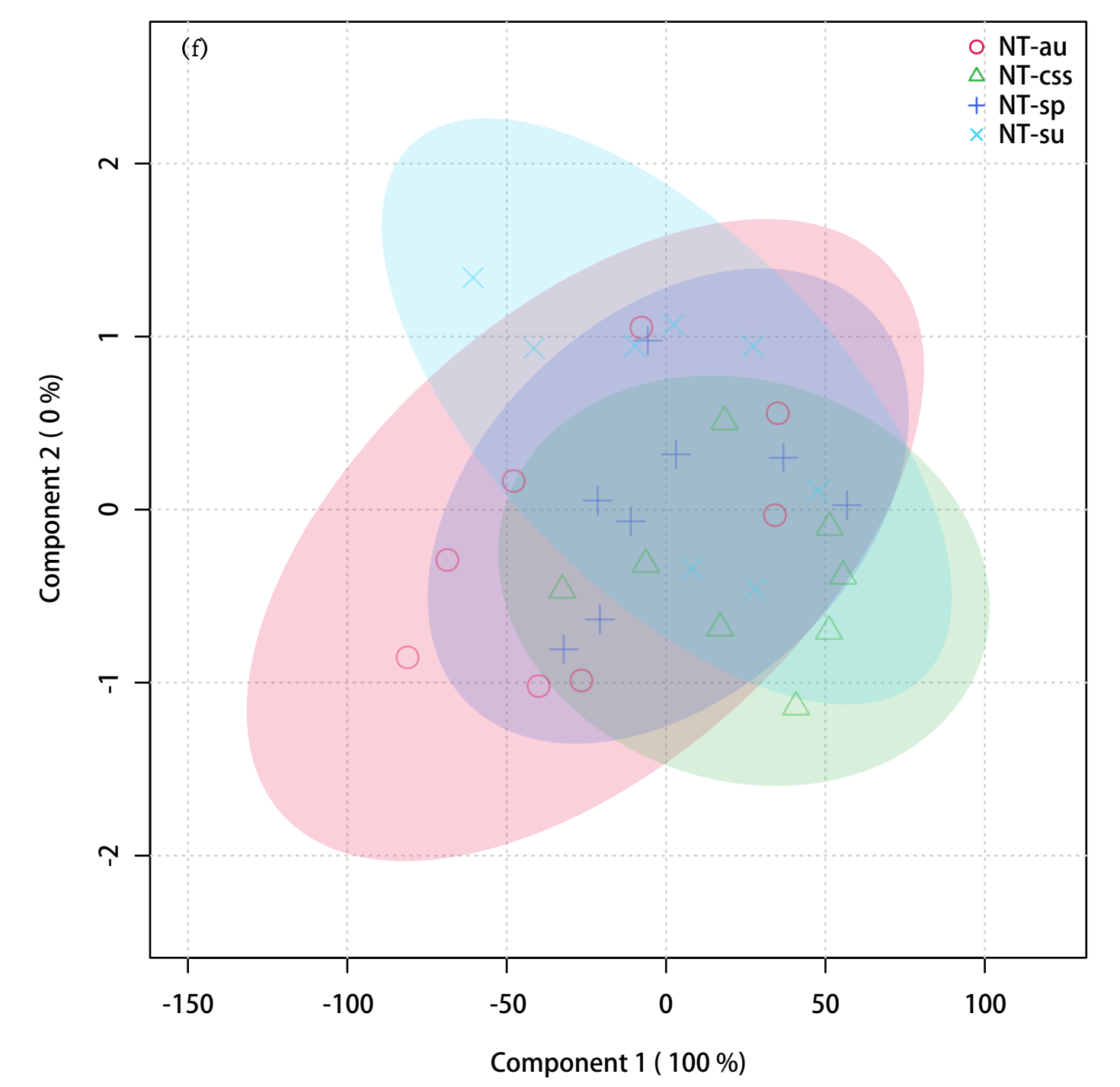

Supplement: Supplementary Figure 1 — Partial least squares-discriminant analysis (PLS-DA) scores of gas exchange parameters of different hybrids in each leaf type and different leaf types in each hybrid. (A–D) show the differences between the two hybrids including OP and NT for each leaf type including current-year spring (css), autumn (au), summer (su), and spring (sp) shoots. (E, F) show the differences between the leaf types for the two hybrids OP and NT. The PLS-DA loading charts contain the parameters Pn (net photosynthesis), Tr (transpiration), Gs (stomatal conductance), Ci (intracellular CO2 concentration), WUEi (intrinsic water use efficiency), and WUEinst (instantaneous water use efficiency). The ellipses indicate the 95% confidence range. [file DataSheet1.pdf]
