# Supplementary material for: Efficient photosynthesis and economic water use of citrus leaves depend on hybrid, cultivar and leaf type
Source: Front Plant Sci. 2025 Sep 4;16:1536703. doi: 10.3389/fpls.2025.1536703 (PMC12443699; doi:10.3389/fpls.2025.1536703)
Supplement: Supplementary Figure 2 — Expression pattern of photosynthesis- and transpiration- related genes. Expression pattern of (A) photosynthesis electron transport-involved genes and (B) participated in the carbon fixation via Calvin-Benson cycle genes and (C) control leaf water status via regulation of stomatal movement. The expression of genes is centered and scaled in row direction. [file DataSheet2.pdf]

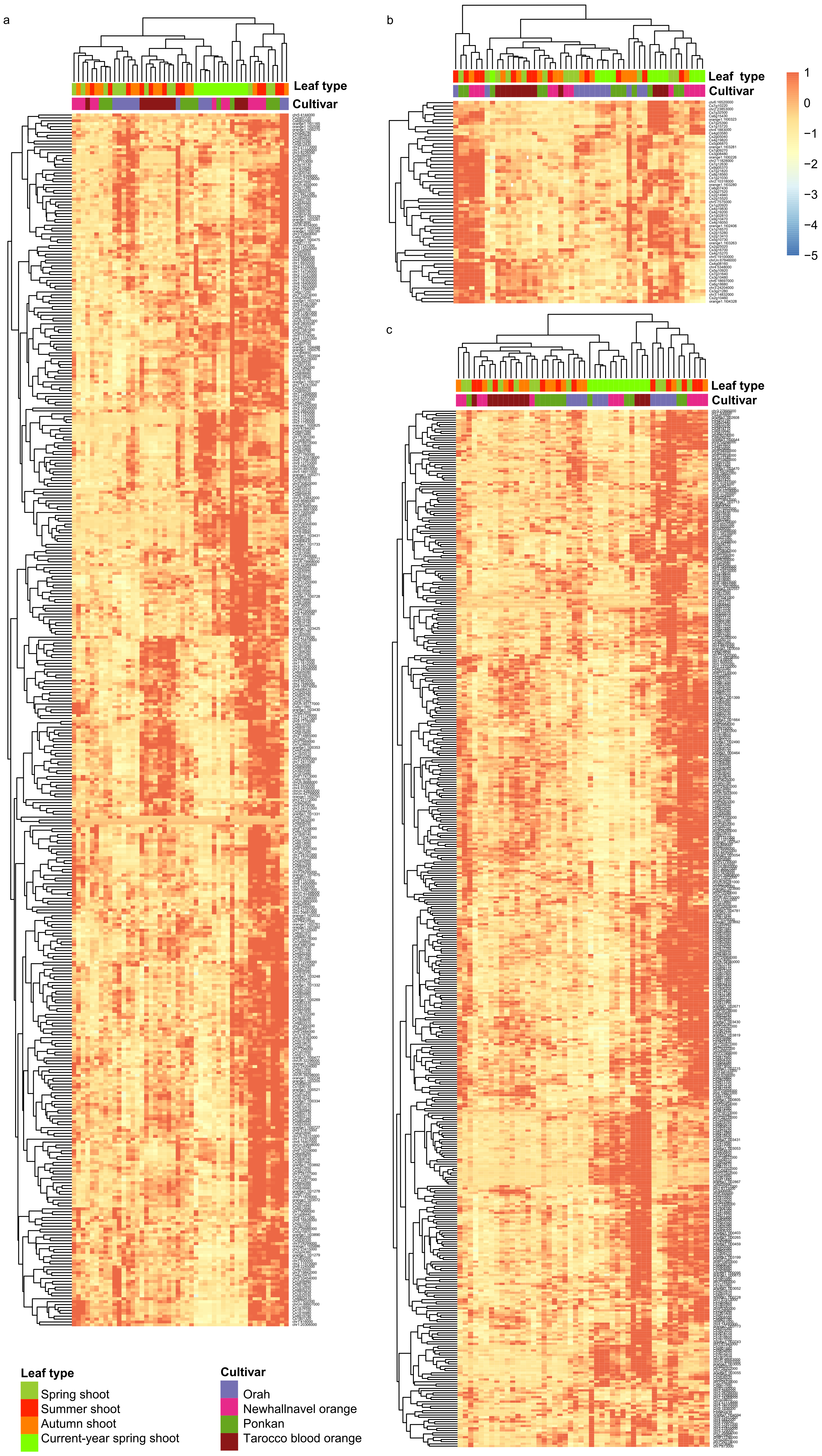
a
b
c
Leaf type
Cultivar
Leaf type
Cultivar
Leaf type
Cultivar
1
0
−1
−2
−3
−4
−5
Leaf type
Spring shoot
Summer shoot
Autumn shoot
Current-year spring shoot
Cultivar
Orah
Newhallnavel orange
Ponkan
Tarocco blood orange
